# Deregulation of CLTC interacts with TFG, facilitating osteosarcoma via the TGF‐beta and AKT/mTOR signaling pathways

**DOI:** 10.1002/ctm2.377

**Published:** 2021-06-20

**Authors:** Li Shijie, Pan Zhen, Qin Kang, Guo Hua, Yang Qingcheng, Cheng Dongdong

**Affiliations:** ^1^ Department of Orthopedics Shanghai Jiao Tong University Affiliated Sixth People's Hospital Shanghai China; ^2^ Department of Trauma and Reconstructive Surgery RWTH Aachen University Hospital Aachen Germany

**Keywords:** CLTC, osteosarocma, proliferation, TFG

## Abstract

Although the treatment of osteosarcoma has improved, the overall survival rate of this common type of osseous malignancies has not changed for four decades. Thus, new targets for better therapeutic regimens are urgently needed. In this study, we found that high expression of clathrin heavy chain (CLTC) was an independent prognostic factor for tumor‐free survival (HzR, 3.049; 95% CI, 1.476–6.301) and overall survival (HzR, 2.469; 95% CI, 1.005–6.067) of patients with osteosarcoma. Down‐regulation of CLTC resulted in tumor‐suppressive effects in vitro and in vivo. Moreover, we found that *CLTC* was transcriptionally regulated by a transcription factor—specificity protein 1 (SP1), which binds to the *CLTC* promoter at the −320 to −314‐nt and +167 to +173‐nt loci. Mechanistic investigations further revealed that CLTC elicited its pro‐tumor effects by directly binding to and stabilizing trafficking from the endoplasmic reticulum to the Golgi regulator (TFG). Importantly, overexpression of TFG rescued both the tumor‐suppressive effect and inhibition of the TGF‐β and AKT/mTOR pathways caused by CLTC down‐regulation, which indicated that the activity of CLTC was TFG‐dependent. Immunohistochemistry analysis confirmed that CLTC expression was positively correlated with TFG expression. These findings collectively highlight CLTC as a new prognostic biomarker for patients with osteosarcoma, and the interruption of the SP1/CLTC/TFG axis may serve as a novel therapeutic strategy for osteosarcoma.

## Graphical abstract

1. High CLTC expression is associated with worse clinical outcomes in osteosarcoma patients.

2. Inhibition of CLTC suppresses osteosarcoma growth both in vitro and in vivo.

3. SP1 binds to the CLTC promoter to promote the transcriptional activity of CLTC in osteosarcoma.

4. CLTC‐mediated oncogenic effects occurred through activation of the TGF‐beta and AKT/mTOR signaling pathway in a TFG‐dependent manner.

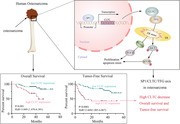

**Abbreviations**
CCK‐8: Cell Counting Kit‐8
ChIP: chromatin immunoprecipitation
CLTC: clathrin heavy chain
COPII: coat protein complex II
ER: endoplasmic reticulum
IHC: immunohistochemistry
IP: immunoprecipitation
SD: standard deviation
SP1: Sp1 transcription factor
TFG: trafficking from ER to Golgi regulator
WB: Western blot

## 1 INTRODUCTION

Osteosarcoma is one of the most common primary osseous malignancies, which is characterized by the direct generation of immature bone and osteoid tissue by tumor cells.^1^ Primary osteosarcomas usually occur in the metaphysis of long bones and have a marked predilection for the knee.^2^ In the early 1970s and with the introduction of adjuvant or neoadjuvant chemotherapy, the long‐term survival rate of patients with osteosarcoma has dramatically improved.^3^ However, the 5‐year overall survival rate of these patients has failed to improve for more than four decades.^4^ This may be attributed to the complex molecular mechanism and extreme heterogeneity of osteosarcoma. Thus, the molecular pathogenesis of osteosarcoma needs to be further explored. Meanwhile, reliable prognostic biomarkers and novel therapeutic targets have to be identified and applied to improve patient survival.

In this study, we collected eight pairs of osteosarcoma tissues and matched normal tissues. For these eight pairs of matched samples, RNA‐sequencing (RNA‐seq) analysis was used to investigate the differentially expressed genes between osteosarcoma tissues and matched normal tissues. Based on the combinatorial analysis of bioinformatics and functional screening, clathrin heavy chain (CLTC) was selected and confirmed as a new biomarker and potential therapeutic target for patients with osteosarcoma. It is a gene encoding clathrin heavy chain on chromosome 17 (CHC17), which participates in the formation of the polyhedral coat of coated pits and vesicles.^5^ Unlike one of the isoforms, clathrin heavy chain on chromosome 22 (CHC22), which is encoded by CLTCL1 and plays a role in the trafficking of insulin‐responsive GLUT4, CLTC is essential for autophagic lysosomal reformation.^6^ In a recent study, CLTC facilitated the fusion of autophagosomes with Coxiella‐containing vacuoles.^7^ As a classical method of endocytosis, clathrin‐mediated endocytosis is crucial for cell biological processes.^8^ In cancer research, CLTC has also been reported to correlate with tumorigenesis. The CLTC–ALK fusion gene has been confirmed to be an ALK activator in large B‐cell lymphoma and is related to tumor recurrence.^9^ This aberrant fusion gene was also considered a primary factor in congenital blastic plasmacytoid dendritic cell neoplasm.^10^ However, the biological function and molecular mechanism of CLTC in osteosarcoma remain unexplored. In this study, we first demonstrated that CLTC was an independent prognostic factor for tumor‐free survival and overall survival of patients with osteosarcoma. Down‐regulation of CLTC inhibited cell proliferation, promoted apoptosis, and blocked the cell cycle transition in osteosarcoma. Subsequent studies demonstrated that Sp1 transcription factor (SP1) binds to the *CLTC* promoter and promotes the transcriptional activity of *CLTC*.

SP1 is a famous member of the transcription factors involved in various essential biological processes and has been proven to play a role in the progression of apoptosis, proliferation, and carcinogenesis.^11^ In breast cancer, SP1 binds to the CD147 promoter and promotes the metastasis of tumor cells by enhancing the transcriptional activity of CD147.^12^ In gastric cancer, it has been documented that SP1 binds to the promoter regions of lncRNA AGAP2‐AS1 and promotes the transcriptional activity of this oncogenic lncRNA.^13^ In this study, we identified that SP1 binds to the *CLTC* promoter at the −320 to −314‐nt and +167 to +173‐nt loci and activates CLTC expression in osteosarcoma cells.

In addition, further mechanical studies suggested that CLTC interacts with TFG and activates the TGF‐β and AKT/mTOR signaling pathways. TFG is also a tumor‐related gene situated on chromosome 3 and is widely expressed in cells.^14^ The function of TFG is to maintain the stability of the endoplasmic reticulum (ER) structure^15^ and is correlated with the protein secretory pathways.^16^ Furthermore, several fusion oncoproteins are encoded partially by TFG, such as TFG‐ALK,^17^ TFG‐RET,^18^ and TFG‐MET.^19^ In this study, we confirmed that TFG was highly expressed in osteosarcoma tissues when compared with matched normal tissues. Down‐regulation of TFG also delayed cell proliferation and promoted apoptosis in osteosarcoma. Moreover, overexpression of TFG rescued the tumor‐suppressive effect and inhibition of the TGF‐β and AKT/mTOR signaling pathways caused by the down‐regulation of CLTC. Immunohistochemistry (IHC) analysis demonstrated that high expression of TFG was also correlated with a poor prognosis in patients with osteosarcoma. In conclusion, our study suggests that SP1 promotes the transcriptional activity of *CLTC*, and CLTC‐mediated oncogenic effects occurred in a TFG‐dependent manner. High expression of CLTC was found to be an independent prognostic factor for tumor‐free survival and overall survival of patients with osteosarcoma.

## 2 RESULTS

### 2.1 CLTC is highly expressed in osteosarcoma and positively correlated with the prognosis of patients with osteosarcoma

**FIGURE 1:**
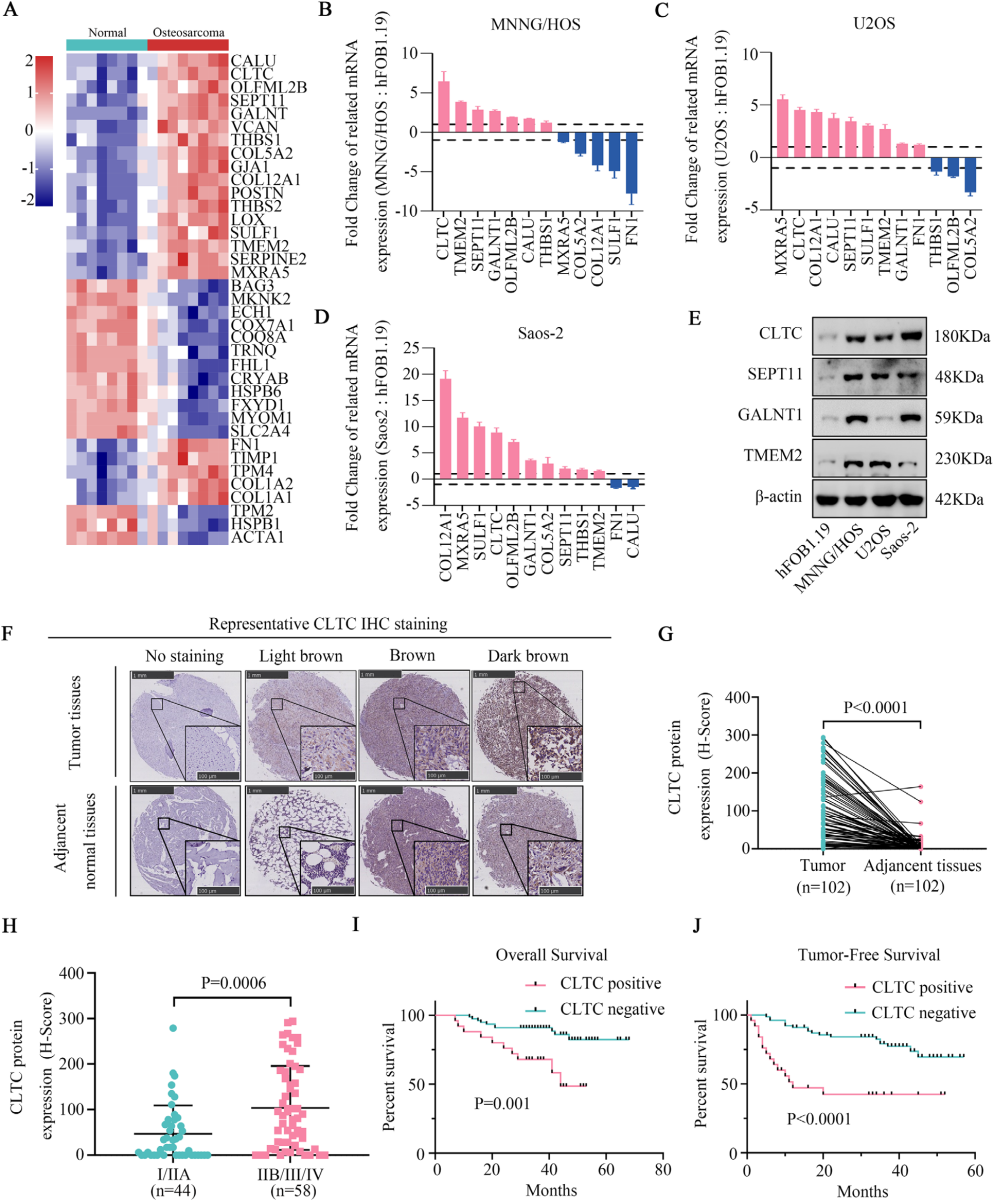
Clathrin heavy chain (CLTC) was highly expressed in osteosarcoma and correlated with a poor prognosis in the patients with osteosarcoma. (A) Heat map of RNA‐seq in eight pairs of osteosarcoma tissues and matched normal tissues. (B) mRNA expression levels of candidate genes in MNNG/HOS and hFOB1.19. (C) mRNA expression levels of candidate genes in U2OS and hFOB1.19. (D) mRNA expression levels of candidate genes in Saos‐2 and hFOB1.19. (E) Protein expression levels of selected genes in hFOB1.19, MNNG/HOS, U2OS, and Saos‐2. (F) Osteosarcoma TMA was used to examine the clinical relevance of CLTC expression levels on the patients’ outcomes. A representative IHC‐stained image is shown (magnification 20×). (G) IHC analysis to quantify the expression of CLTC in osteosarcoma tissues and matched adjacent normal tissues. (H) IHC analysis to quantify the expression of CLTC in patients with clinical early stage (I/IIA) versus advanced stage (IIB/III/IV) tumor. (I) Kaplan–Meier analysis was used to determine the overall survival of CLTC‐positive and CLTC‐negative patients. (J) Kaplan–Meier analysis was used to determine the tumor‐free survival of CLTC‐positive and CLTC‐negative patients. Error bars represent the standard deviation (SD). *p < .05; **p < .01

To study the molecular pathogenesis of osteosarcoma, we subjected eight pairs of osteosarcoma tissues and matched normal tissues to RNA‐seq analysis. In the bioinformatics analysis, 37 genes were found to be significantly differentially expressed more than two‐fold between all eight pairs of osteosarcoma tissues and matched normal tissues, including 22 up‐regulated genes and 15 down‐regulated genes. The heat map of these dysregulated genes is shown in Figure 1A. Gene ontology (GO) analysis and Kyoto Encyclopedia of Genes and Genomes (KEGG) pathway analysis were performed on the dysregulated genes, which showed the potential biological processes involved in the tumorigenesis of osteosarcoma (Figure S1). To screen for tumorigenic factors, we focused on the up‐regulated genes. Based on the PubMed data, 10 out of the 22 up‐regulated genes (*VCAN*, *TPM4*, *GJA1*, *POSTN*, *THBS2*, *LOX*, *SERPINE2*, *TIMP1*, *COL1A1*, and *COL1A2*) have been reported to be associated with osteosarcoma. Therefore, the remaining candidate genes (*CLTC*, *TMEM2*, *SEPT11*, *GALNT1*, *OLFML2B*, *CALU*, *THBS1*, *MXRA5*, *COL5A2*, *COL12A1*, *SULF1*, and *FN1*) were selected for further study. To investigate the role of these 12 genes, we first validated the mRNA expression of these genes in osteosarcoma and osteoblast cell lines. Four out of the 12 genes (*CLTC*, *GALNT1*, *SEPT11*, and *TMEM2*) were confirmed to have a relatively high expression in MNNG/HOS, U2OS, and Saos‐2 cell lines in comparison to that in a healthy osteoblast control cell line hFOB1.19 (Figure 1B–D). The high mRNA expression of these genes was further demonstrated in the eight pairs of osteosarcoma tissues in comparison to that in matched normal tissues (Figure S2). Moreover, both CLTC and SEPT11 were found to have consistently high expression in all osteosarcoma cells when compared to those in the osteoblast cells; conversely, the other two genes (GALNT1 and TMEM2) were not (Figure 1E). The consistently high expression of CLTC and SEPT11 indicated that they might contribute to the tumorigenesis of osteosarcoma. To confirm whether CLTC and SEPT11 were proliferation‐related genes, we used the Cell Counting Kit‐8 (CCK‐8) assay for functional screening. The analysis demonstrated that down‐regulation of CLTC dramatically attenuated cell proliferation in MNNG/HOS, but that of SEPT11 did not (Figure S3).

Thus, we investigated the clinical significance of CLTC. We detected and compared CLTC expression via IHC analysis in osteosarcoma TMA, which contained 102 pairs of matched osteosarcoma and normal tissues. Representative IHC staining results are shown in Figure 1F. The IHC results (H score) indicated that CLTC expression was significantly higher in the osteosarcoma tissues than in the matched normal tissues (Figure 1G). Clinical analysis showed that the expression of CLTC was positively correlated to the AJCC/TNM stage and recurrence rate of osteosarcoma (Figure 1H, Table 1), while no significant correlation was found in gender, age, tumor location, operation type, and tumor necrosis rate (Table 1). In addition, the Kaplan–Meier survival assay showed that the patients with positive CLTC expression had a shorter tumor‐free and overall survival than the patients with negative CLTC expression (Figure 1I,J). Importantly, the univariate and multivariate Cox proportional hazards analysis findings suggested that CLTC was an independent prognostic variable for tumor‐free survival (HzR, 3.049; 95% CI, 1.476–6.301) and overall survival (HzR, 2.469; 95% CI, 1.005–6.067) of patients with osteosarcoma (Table 2). Collectively, these findings suggested that CLTC is highly expressed in osteosarcoma and is a valuable prognostic factor for patients with osteosarcoma.

**TABLE 1:** Correlation analyses of clathrin heavy chain (CLTC) protein expression in relation to clinicopathologic variables of 102 patients with osteosarcoma

Clinicopathologic parameters	CLTC expression level		*p*‐Value
	Negative	Positive	
Gender			
Male	24	11	.24
Female	53	14	
Age (years)			
<20	43	18	.152
≥20	34	7	
Location			
Distal femur	41	14	.716
Proximal tibia	18	4	
Elsewhere	18	7	
Tumor necrosis rate (%)			
<90	59	18	0.641
≥90	18	7	
Operation type			
Amputation	10	6	.188
Limb salvage	67	19	
Recurrence			
Yes	8	12	.000*
No	69	13	
AJCC/TNM stage			
I/IIA	40	4	.002*
IIB/III/IV	37	21	

^*^ *P* < 0.05.

**TABLE 2:** Variables predictive of survival by Cox proportional hazards model in osteosarcoma

Variables		No.	Disease‐free survival		Overall survival		
Univariate analysis			*p*‐Value	Hazard ratio (95% confidence interval)	*p*‐Value	Hazard ratio (95% confidence interval)	
	Gender						
	Male	35	.582	1.242 (0.574–2.684)	.836	1.106 (0.425–2.884)	
	Female	67					
	Age (years)						
	≤20	61	.055	0.457 (0.205–1.017)	.289	0.596 (0.229–1.552)	
	>20	41					
	Location						
	Distal femur	55	.631	1.104 (0.737–1.652)	.687	1.111 (0.667–1.850)	
	Proximal tibia	22					
	Elsewhere	25					
	Tumor necrosis rate (%)						
	<90	77	.131	2.092 (0.803–5.450)	.05	7.505 (1.003–56.153)	
	≥90	25					
	Operation type						
	Amputation	16	.202	1.729 (0.746–4.009)	.056	2.546 (0.978–6.629)	
	Limb salvage	86					
	AJCC/TNM stage						
	I/IIA	56	.001*	4.660 (1.906–11.391)	.005*	17.579 (2.351–131.433)	
	IIB/III/IV	46					
	CLTC						
	positive	25	.000*	4.082 (2.012–8.281)	.002*	3.928 (1.625–9.495)	
	negative	77					

Multivariate analysis	Variables	Wald χ^2^	*p*‐Value	Hazard ratio (95% confidence interval)	Wald χ^2^	*p*‐Value	Hazard ratio (95% confidence interval)
	AJCC/TNM stage	7.922	.005*	3.735 (1.492–9.350)	6.476	.011*	13.968 (1.833–106.430)
	CLTC	9.065	.003*	3.049 (1.476–6.301)	3.886	.049*	2.469 (1.005–6.067)

^*^ *P* < 0.05.

### 2.2 Down‐regulation of CLTC has a tumor‐suppressive effect on osteosarcoma cells both in vitro and in vivo

**FIGURE 2:**
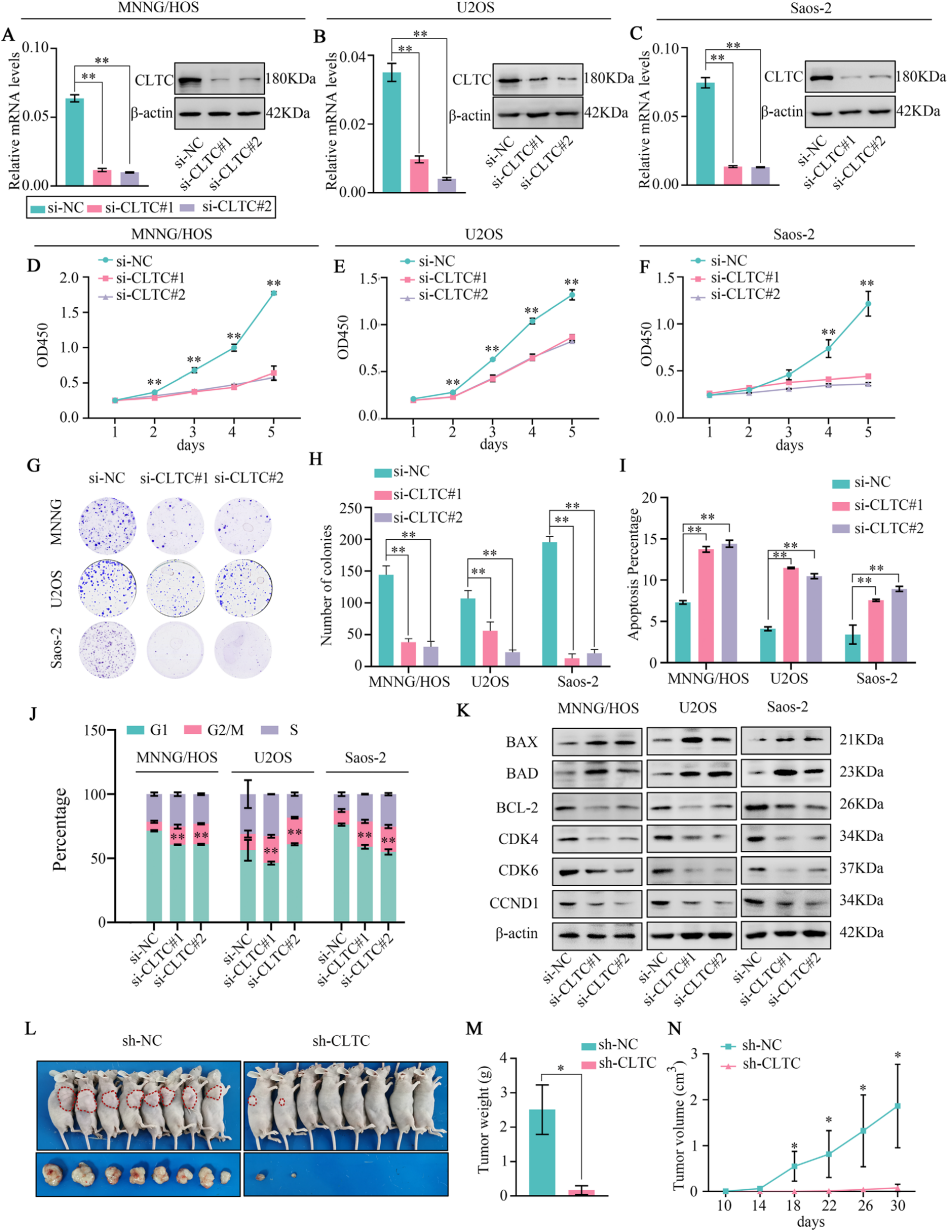
Effects of knockdown of clathrin heavy chain (CLTC) on osteosarcoma cell proliferation, apoptosis, and cell cycle. (A–C) After transfection of two independent CLTC siRNAs in MNNG/HOS, U2OS, and Saos‐2, the levels of mRNA and protein were verified. (D–F) CCK‐8 assay was performed for CLTC knockdown osteosarcoma cells and control cells. (G and H) Colony formation assay for CLTC knockdown osteosarcoma cells and control cells. (I) Apoptosis assay for CLTC knockdown osteosarcoma cells and control cells. (J) Cell cycle assay for CLTC knockdown osteosarcoma cells and control cells. (K) WB was used to detect the apoptotic and cell cycle‐related markers in the MNNG/HOS, U2OS, and Saos‐2 cells. (L) Xenograft tumors formed by the sh‐CLTC targeted MNNG/HOS cells and control cells. (M) Weight of the xenograft tumors in sh‐NC and sh‐CLTC groups. (N) Growth curve demonstrating the tumor volumes on indicated days. Error bars represent the standard deviation (SD). *p < .05; **p < .01

To investigate the function of CLTC in osteosarcoma cells, we utilized two independent siRNAs to down‐regulate the expression of *CLTC*. Western blotting (WB) and quantitative real‐time PCR (qRT‐PCR) assays showed that both CLTC siRNAs significantly decreased CLTC expression in the osteosarcoma cell lines (Figure 2A–C). In the CCK‐8 assay, we observed a significant reduction in the cells transduced with the two CLTC siRNAs. Similar results were obtained for all three osteosarcoma cell lines (Figure 2D–F). The colony formation assay demonstrated that down‐regulation of CLTC attenuated the ability of osteosarcoma cells to form colonies (Figure 2G,H). However, the migration ability of the osteosarcoma cells was not affected by the expression of CLTC (Figure S4). Moreover, we observed that CLTC knockdown promoted apoptosis in the osteosarcoma cells (Figure 2I and Figure S5A). The cell cycle assay findings suggested that delayed cell proliferation might be linked to cell cycle arrest. Studies demonstrated that the down‐regulation of CLTC arrested cell cycle progression in the G2/M phase (Figure 2J and Figure S5B). We evaluated the effects of CLTC knockdown in a healthy osteoblast control cell line hFOB1.19, and the general cytotoxicity upon CLTC knockdown was not observed in hFOB1.19 cells (Figure S6). Furthermore, we detected changes in the protein levels of apoptosis and cell cycle‐related markers after CLTC knockdown. Down‐regulation of CLTC significantly attenuated the expression of BCL‐2, CDK4, CDK6, and CCND1 and promoted the expression of BAX and BAD (Figure 2K). These results supported that down‐regulation of CLTC promoted apoptosis and arrested cell cycle in the osteosarcoma cells. To test whether these tumor‐suppressive effects were not attributed to the loss of clathrin function, we knocked down the expression of CLTCL1, which is another isoform of clathrin heavy chain. We found that down‐regulation of CLTCL1 did not impair cell growth in osteosarcoma (Figure S7). These results show that the tumor‐suppressive effect of CLTC knockdown was CLTC‐specific.

To further investigate the effect of CLTC in vivo, MNNG/HOS were stably transfected with shRNA‐CLTC (Figure S8A,B). WB and qRT‐PCR assay showed that CLTC sh‐RNA significantly decreased CLTC expression in MNNG/HOS cells (Figure S8A,B). A xenograft model experiment was used to assess the tumorigenesis role of CLTC in vivo. The analysis showed a significant decrease in tumorigenesis in the sh‐CLTC group when compared to that in the sh‐NC group (Figure 2L). Meanwhile, the tumor growth rate was lower in sh‐CLTC group with smaller tumor volumes and smaller tumor weights than in the sh‐NC group (Figure 2M,N). In terms of mouse body weight, there was no significant difference between the two groups (Figure S8C). Furthermore, IHC staining for CLTC and Ki67 (a tissue proliferation marker) showed simultaneous lighter staining in the sh‐CLTC xenografts than in the sh‐NC xenografts (Figure S8D). These results collectively supported that CLTC knockdown inhibited tumor growth in vivo. Taken together, CLTC knockdown had a tumor‐suppressive effect in osteosarcoma both in vitro and in vivo.

### 2.3 SP1 targets the CLTC promoter and induces high expression of CLTC in osteosarcoma

**FIGURE 3:**
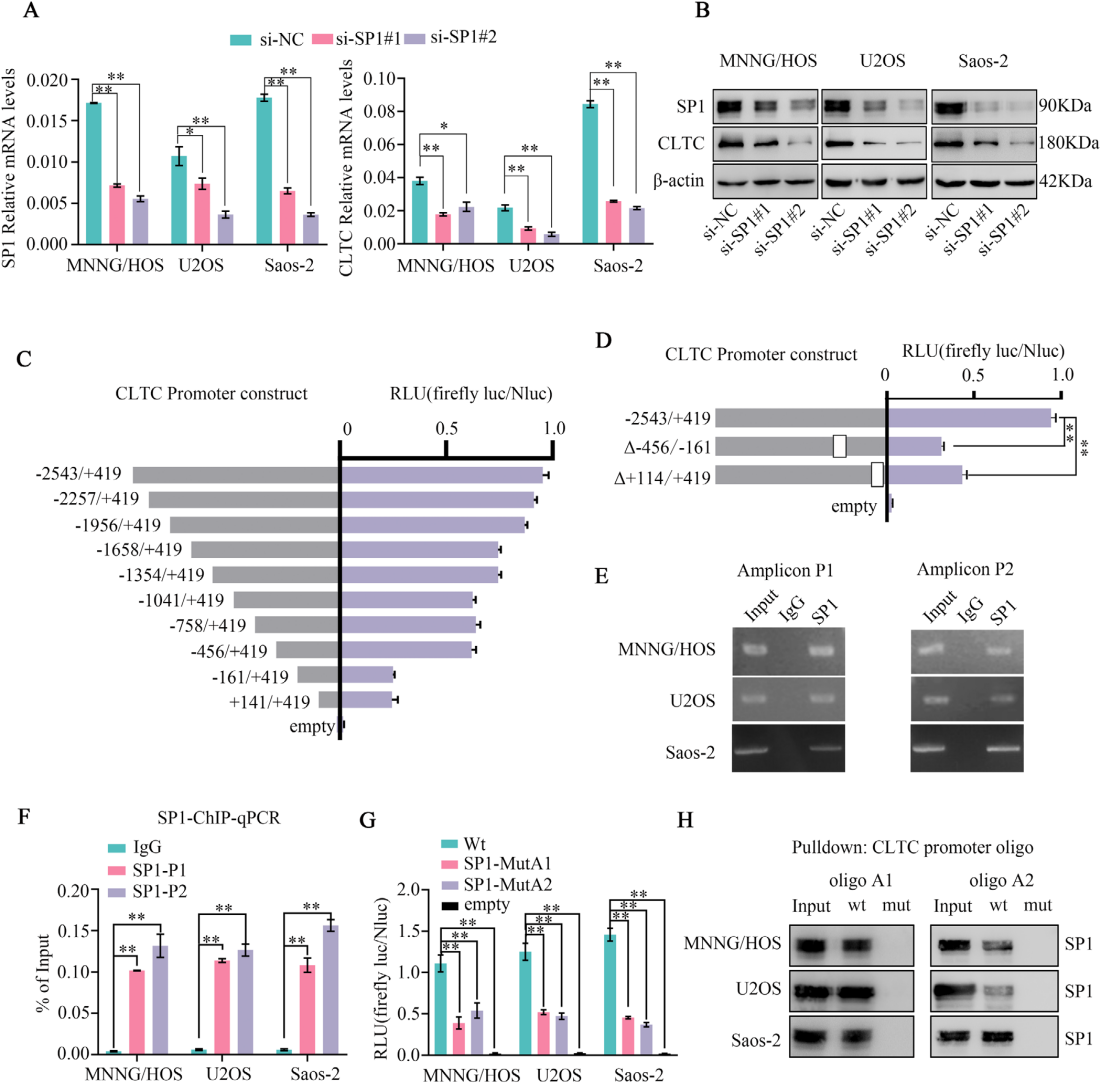
SP1 directly bound on the clathrin heavy chain (CLTC) promoter and promoted the transcriptional activity of CLTC. (A and B) Down‐regulation of SP1 reduced the expression of CLTC in mRNA and protein levels. (C) Luciferase reporter gene plasmids of the CLTC promoter sequence and serial deletion mutant were constructed. MNNG/HOS cells were transfected with the indicated plasmids, and the luciferase activities were determined using a dual luciferase assay. (D) Luciferase assay results for the CLTC promoter constructs in which the −456 to −161‐nt or +114 to +419‐nt segments were deleted from the full‐length CLTC promoter. (E and F) ChIP analysis of SP1 was performed followed by PCR amplification of the SP1 gene locus P1 (−456 to −161 nt) and P2 (+114 to +419 nt). (G) Putative SP1 binding sites A1 (−320 to −314 nt) and A2 (+167 to +173 nt) mutation luciferase reporter gene plasmids were constructed. The luciferase activity of the reporter plasmids carrying wild‐type, mutant‐type A1, or mutant‐type A2 in the osteosarcoma cells was detected. (H) DNA pull‐down assay was used to detect SP1 binding by the −320 to −314‐nt (Oligo A1) and +167 to +173‐nt (Oligo A2) SP1 binding sites on the CLTC promoter sequence

The transcription factor is of significance in tuning the expression of protein‐coding genes. Previous studies have suggested that transcription factors OCT‐1, AP1, SP1, TNF, SOX2, SOX4, and MYC play an important role in the proliferation of osteosarcoma cells.^20^, ^21^, ^22^, ^23^ To study whether these proliferation‐related transcription factors correlated with CLTC, we down‐regulated the expression of OCT‐1, AP1, SP1, TNF, SOX2, SOX4, and MYC in the MNNG/HOS cells. We found down‐regulation of the SP1 decreased the mRNA and protein levels of CLTC (Figure 3A,B). The expression of CLTC was unchanged when other transcription factors were knocked down (Figure S9). Considering that down‐regulation of SP1 resulted in a significant decrease in CLTC expression, it was reasonable to speculate that SP1 targeted the *CLTC* promoter and thus promoted the transcriptional activity of *CLTC*. To verify this speculation and investigate the crucial binding sites of SP1 on *the CLTC* promoter, we constructed luciferase reporter gene plasmids with the entire *CLTC* promoter sequence and the *CLTC* promoter serial deletion mutants. The luciferase assay showed that the transcriptional activity of *CLTC* decreased significantly in the MNNG/HOS cells when −456 to −161 nt or +141 to +419 nt was truncated (Figure 3C). To further confirm whether these two promoter segments were truly important for *CLTC* regulation, we constructed and transfected luciferase reporter gene plasmids lacking −456 to −161 nt or +141 to +419 nt fragmented *CLTC* promoters into the MNNG/HOS cells. The analysis demonstrated that the deletion of these fragments (−456 to −161 nt or +141 to +419 nt) both resulted in a significant decrease in the transcriptional activity of *CLTC* (Figure 3D). Next, chromatin immunoprecipitation (ChIP) and ChIP‐PCR assays were performed to confirm the binding of SP1 to the P1 (−456 to −161 nt) and P2 (+141 to +419 nt) regions on *the CLTC* promoter (Figure 3E,F). Thereafter, online databases were used to predict the most likely SP1 binding sites in the P1 (−456 to −161 nt) and P2 (+141 to +419 nt) regions on *the CLTC* promoter. Two SP1 potential binding sites, that is, A1 (−320 to −314 nt) and A2 (+167 to +173 nt), on *the CLTC* promoter were predicted on the basis of the online database JASPAR (http://jaspar.genereg.net/). Thus, the corresponding binding site mutants were constructed. Intriguingly, the luciferase assay showed that the transcriptional activity of *CLTC* was significantly reduced when the predicted SP1 binding sites on the CLTC promoter were mutated (Figure 3G). To further confirm this conclusion, we synthesized biotinylated double‐stranded DNAs containing SP1 binding loci (A1 or A2) and the corresponding mutant biotinylated DNAs. WB of the DNA binding assay also confirmed the binding of SP1 to the A1 (−320 to −314 nt) and A2 (+167 to +173 nt) loci on the *CLTC* promoter (Figure 3H). These results collectively suggested that SP1 physically binds to the *CLTC* promoter at the −320 to −314‐nt and +167 to +173‐nt loci to promote the transcriptional activity of CLTC in osteosarcoma. Down‐regulation of SP1 inhibited cell proliferation in osteosarcoma (Figure S10). This inhibition can be reversed by overexpression of CLTC (Figure S11).

### 2.4 CLTC functions as an oncogene via the TGF‐β and AKT/mTOR signaling pathways

**FIGURE 4:**
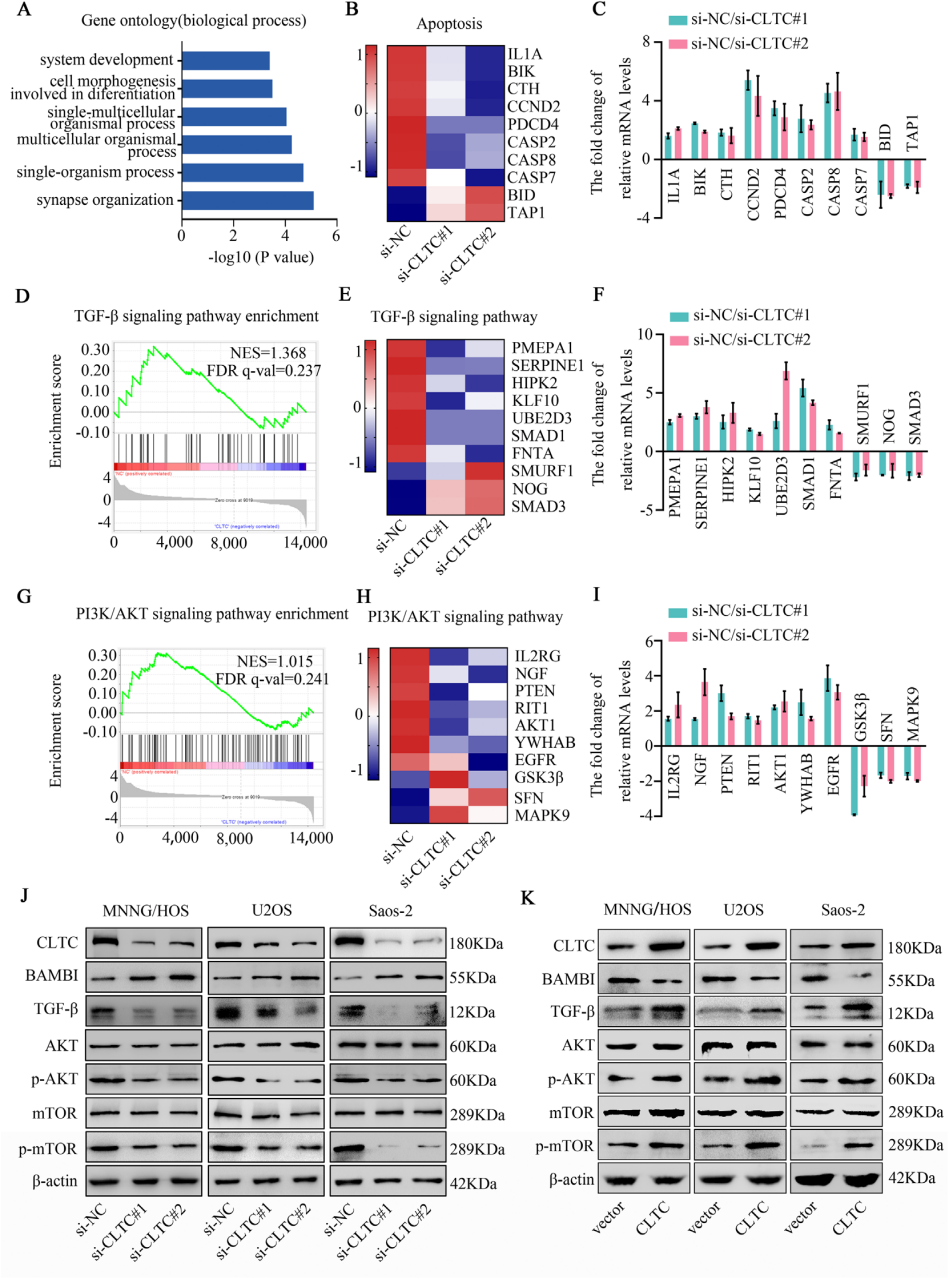
Clathrin heavy chain (CLTC) regulates the TGF‐Î² and AKT/mTOR signaling pathways in osteosarcoma. (A) GO analysis was used to analyze the functional annotation clustering of genes regulated by the down‐regulation of CLTC in MNNG/HOS. (B) The apoptosis subset was enriched in GSEA, and the heat map shows the expression levels of selected genes. Blue, white, and red shadings in the heat map indicate low, intermediate, or high gene expressions, respectively. (C) Gene expression of selected genes from the apoptosis subset detected by qRT‐PCR assay in MNNG/HOS. (D–F) The TGF‐β signaling pathway was enriched in GSEA (D). The heat map shows the gene expression levels of the subset (E). The qRT‐PCR analysis confirmed the expression changes in the genes selected from the subgroup in MNNG/HOS (F). (G–I) The PI3K/AKT/mTOR signaling pathway was enriched in GSEA (G). The heat map shows the gene expression levels of the subset (H). The qRT‐PCR analysis confirmed the expression changes in the genes selected from the subgroup in MNNG/HOS (I). (J) Representative blots show the protein levels of BAMBI, TGF‐β, AKT, p‐AKT, mTOR, and p‐mTOR in the MNNG/HOS, U2OS, and Saos‐2 cells after transfection by si‐CLTCs. β‐actin was used as an internal control. (K) Representative blots show the protein levels of BAMBI, TGF‐β, AKT, p‐AKT, mTOR, and p‐mTOR in osteosarcoma cells following overexpression of CLTC. β‐actin served as the internal control. Statistical analysis was performed using Student's *t*‐test

To investigate the role of CLTC, RNA‐seq analysis was utilized to elucidate the total transcriptional changes in the MNNG/HOS cells after the down‐regulation of CLTC expression. The GO analysis of RNA‐seq indicated that CLTC affected many biological processes, such as synapse organization, single‐organism process, and multicellular organismal process (Figure 4A). Gene set enrichment analysis (GSEA) was utilized to reveal the gene signature regulated by CLTC. The process of apoptosis (NES = 1.067, FDR *q*‐value = 0.215) was enriched by GSEA (Figure 4B). The results of the qRT‐PCR experiments confirmed that apoptotic‐related markers were significantly affected after interfering with the expression of CLTC (Figure 4C). In line with our findings, these results also supported that the apoptosis of osteosarcoma cells was affected by the expression of CLTC. Moreover, the TGF‐β (NES = 1.368, FDR *q*‐value = 0.237) (Figure 4D,E) and PI3k/AKT/mTOR signaling pathways (NES = 1.015, FDR *q*‐value = 0.241) (Figure 4G,H) were significantly enriched in GSEA, which suggested that they might play crucial roles in the down‐regulation of CLTC. The qRT‐PCR experiment further confirmed alterations in the top‐scoring genes in the TGF‐β and PI3k/AKT/mTOR signaling pathways following CLTC knockdown (Figure 4F,I). To investigate whether these signaling pathways were regulated by CLTC, we performed WB to detect changes in the expression of key node proteins in the TGF‐β and AKT/mTOR signaling pathways after CLTC knockdown. The down‐regulation of CLTC inhibited the phosphorylation of AKT and mTOR, and the total amount of TGF‐β. As a negative regulator of the TGF‐β signaling pathway, BAMBI was up‐regulated by CLTC knockdown. Meanwhile, the total AKT and mTOR protein levels were not significantly altered (Figure 4J). We then collected xenograft tumors from the xenograft model experiment, which was mentioned above. The protein levels of the TGF‐β and PI3k/AKT/mTOR signaling pathways were detected via WB and IHC staining. Compared with the sh‐NC group, TGF‐β and AKT/mTOR signaling pathways were inhibited in the sh‐CLTC group (Figures S12 and S13). Overexpression of CLTC up‐regulated the expression of p‐AKT, p‐mTOR, and TGF‐β and down‐regulated the expression of BAMBI (Figure 4K). Our experiments also suggested that both CDK4/6 inhibitor Palbociclib and AKT inhibitor Perifosine have an antiproliferation effect on osteosarcoma in vitro (Figure S14A). Perifosine but not Palbociclib had a pro‐apoptotic effect on osteosarcoma cells (Figure S14B). Meanwhile, we used Palbociclib and Perifosine to treat tumor‐bearing mice and found that both drugs inhibited tumor proliferation in vivo, in which AKT inhibitor Perifosine promoted apoptosis of osteosarcoma cells in vivo (Figure S15). The concomitant use of both inhibitors also has antiproliferative and pro‐apoptotic effects in osteosarcoma in vivo and in vitro, but the additive effect of Palbociclib and Perifosine on tumor suppressing was not observed in the experiments (Figures S14 and S15). These data revealed that CLTC functioned as an oncogene by regulating the TGF‐beta and AKT/mTOR signaling pathways, and the results encourage the development of CDK4/6 inhibitors and AKT inhibitors for osteosarcoma therapy.

### 2.5 CLTC interacts with TFG and stabilizes TFG in osteosarcoma cells

**FIGURE 5:**
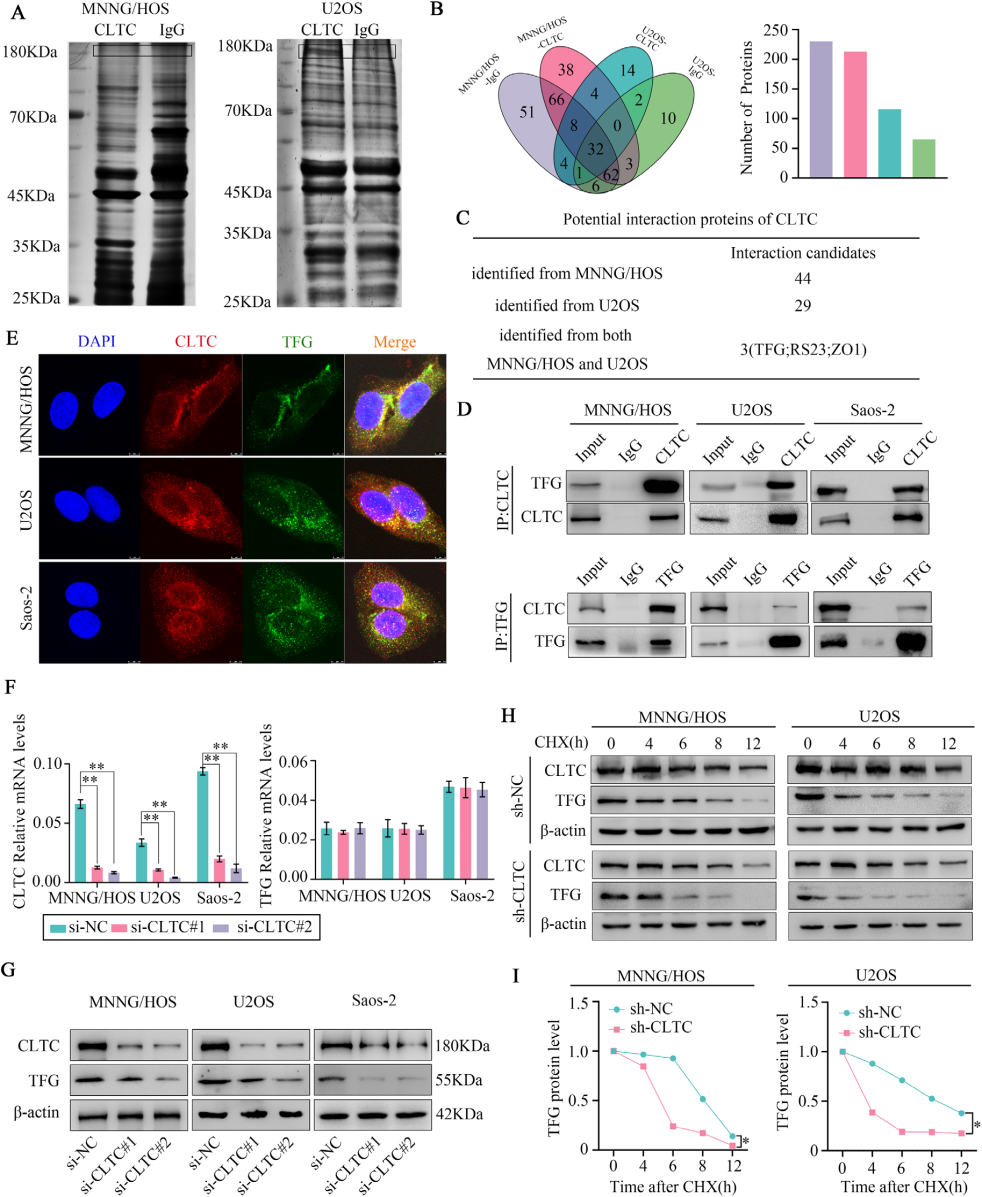
Clathrin heavy chain (CLTC) interacted with TFG and stabilized the TFG protein. (A) Co‐IP assay was employed to determine the protein interaction with CLTC. Immunoprecipitate isolated by the CLTC antibody was detected in sodium dodecyl sulfate polyacrylamide gel electrophoresis (SDS‐PAGE), followed by silver staining. (B) Distribution of the protein numbers identified in MS analysis in the MNNG‐CLTC, MNNG‐IgG, U2OS‐CLTC, and U2OS‐IgG groups. (C) Number of potential interaction proteins of CLTC identified in the MNNG/HOS and U2OS cells. (D) Co‐IP was conducted using an anti‐CLTC antibody (upper diagram) or anti‐TFG antibody (lower diagram). The immunoblotting assay detected anti‐CLTC and anti‐TFG antibodies, respectively. IgG was used as a negative control. (E) Immunofluorescence experiments confirmed the presence of CLTC and co‐localized TFG. CLTC was labeled using red fluorescence, TFG using green fluorescence, and the nucleus using blue fluorescence. (F) After CLTC knockdown, there was no significant change found in TFG expression at the mRNA level. (G) After CLTC knockdown, there was a significant decrease observed in TFG expression at the protein level. (H and I) The MNNG/HOS and U2OS cells with or without CLTC knockdown underwent cyclohexane (100 μg/ml) treatment at 0, 4, 8, 12, and 16 h. The cell lysates were examined via WB (H). A plot of the normalized amount of TFG protein is shown. The quantification of TFG levels is relative to β‐actin, which then normalized to the time = 0 (I)

To further study the mechanism of CLTC, we used co‐immunoprecipitation (Co‐IP) and mass spectrometry (MS) analyses to identify the interacting proteins of CLTC. The immunoprecipitation (IP) samples of CLTC were examined via sodium dodecyl sulfate polyacrylamide gel electrophoresis (SDS‐PAGE) followed by silver staining (Figure 5A). Thereafter, the IP samples had undergone in‐gel trypsin digestion and subjected to MS analysis. In the MNNG/HOS cells, 213 proteins were identified in the CLTC group and 230 proteins in the IgG group after filtering for a peptide number of ≥2. In the U2OS cells, 65 and 113 proteins were identified in the CLTC and IgG groups, respectively, after filtering out proteins with a peptide number of ≥2. As shown in the Venn diagram in Figure 5B, there was an overlap in protein identifications between the two groups. In the MS analysis, 44 and 29 candidate proteins were respectively identified to potentially interact with CLTC in the U2OS and MNNG/HOS cells. Three proteins (TFG, ZO1, and RS23) were found in both U2OS and MNNG/HOS cells (Figure 5C). To confirm whether these three proteins interacted with CLTC, Co‐IP‐WB was employed for further study. The results confirmed that ZO1 and RS23 did not interact with CLTC (Figure S16A). However, CLTC was found to interact with TFG in the CLTC pull‐down experiment (Figure 5D). Reciprocal IP was conducted to further confirmed the interaction between TFG and CLTC (Figure 5D). Meanwhile, the immunofluorescence (IF) assay showed positive co‐localization between CLTC and TFG in the osteosarcoma cells (Figure 5E). This evidence collectively suggested that there was an endogenous interaction between CLTC and TFG. To further study the mechanism of the interaction between CLTC and TFG, we interfered with the expression of CLTC and TFG. The analysis showed a significant decrease in the TFG protein levels when CLTC was down‐regulated, while the mRNA expression of TFG remained unchanged (Figure 5F,G). Moreover, the expression of CLTC was unchanged after TFG knockdown (Figure S16B,C). These results demonstrated that TFG, as a downstream protein, was regulated by CLTC. To further confirm whether CLTC affected the protein stability of TFG, we measured the TFG half‐life using cyclohexane (CHX) chase assays. The results showed that CLTC knockdown significantly decreased the half‐life of TFG in the osteosarcoma cells (Figure 5H,I). In conclusion, these results confirmed that CLTC interacts with TFG in osteosarcoma cells and promotes the protein stabilization of TFG.

### 2.6 The oncogene TFG is essential for CLTC‐mediated osteosarcoma progression

**FIGURE 6:**
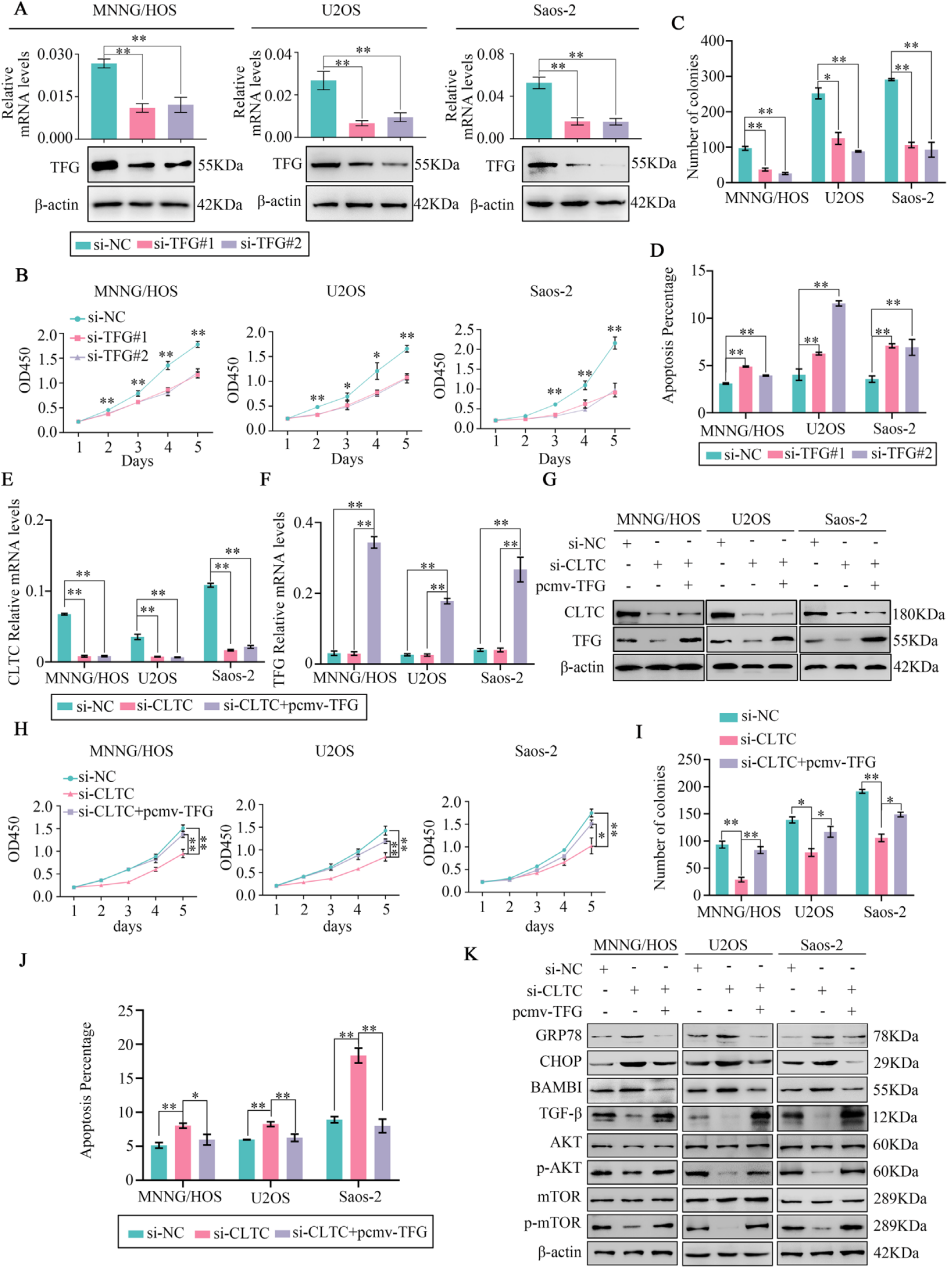
Overexpression of TFG rescued the tumor‐suppressive effect of clathrin heavy chain (CLTC) knockdown in the osteosarcoma cells. (A) After transfection of two independent TFG siRNAs in the MNNG/HOS, U2OS, and Saos‐2 cells, the levels of mRNA and protein were verified. (B) CCK‐8 assay was performed in the MNNG/HOS, U2OS, and Saos‐2 cells after TFG knockdown. (C) Colony formation assay was performed in the MNNG/HOS, U2OS, and Saos‐2 cells after si‐TFG transfection. (D) Flow cytometry was employed to detect the apoptosis percentages of the TFG‐silence cells and control cells. (E and F) qRT‐PCR assay showing the CLTC and TFG expression in osteosarcoma cells transfected with si‐NC and si‐CLTC and co‐transfected with si‐CLTC and pCMV‐TFG. (G) WB showing CLTC and TFG protein expression in the osteosarcoma cells transfected with si‐NC and si‐CLTC and co‐transfected with si‐CLTC and pCMV‐TFG. (H) CCK‐8 assays were used to determine the cell proliferation for osteosarcoma cells transfected with si‐NC and si‐CLTC and co‐transfected with si‐CLTC and pCMV‐TFG. (I) Colony formation assays for the osteosarcoma cells transfected with si‐NC and si‐CLTC and co‐transfected with si‐CLTC and pCMV‐TFG. (J) Flow cytometry was employed to detect the apoptosis rate of osteosarcoma cells transfected with si‐NC and si‐CLTC and co‐transfected with si‐CLTC and pCMV‐TFG. (K) Representative blots of GRP78, CHOP, BAMBI, TGF‐β, AKT, p‐AKT, mTOR, and p‐mTOR in MNNG/HOS, U2OS, and Saos‐2 cells after transfection with si‐NC and si‐CLTC and co‐transfection with si‐CLTC and pCMV‐TFG. β‐actin was used as an internal control. Values represent the mean ± SD from three independent experiments. *p < .05; **p < .01

To better understand the role of TFG in CLTC‐mediated osteosarcoma progression, we first investigated the function of TFG. Two specific siRNAs were utilized to inhibit the expression of TFG in the osteosarcoma cells. WB and qRT‐PCR assay showed that TFG siRNAs significantly decreased TFG expression in the osteosarcoma cell lines (Figure 6A). CCK‐8, colony formation, and apoptosis assays confirmed that TFG knockdown attenuated cell proliferation and promoted apoptosis in the osteosarcoma cells (Figure 6B–D, Figure S17A,B). These results suggested that TFG plays an oncogenic role in osteosarcoma. SEC24A and SEC31A are both components of coat protein complex II (COPII), which is required in the process of ER to Golgi protein trafficking.^24^, ^25^ Down‐regulation of CLTC inhibited the expression of SEC24A and SEC31A (Figure S18). These results indicated that ER to Golgi protein trafficking was regulated by CLTC. Meanwhile, down‐regulation of both CLTC and TFG up‐regulated the expression of GRP78 and CHOP (Figure S19). These results suggested that knockdown of CLTC and TFG could activate ER stress. Considering that TFG is a downstream protein of CLTC and has an oncogenic effect, we next hypothesized that CLTC functions in a TFG‐dependent manner in osteosarcoma. To confirm this hypothesis, we established and transfected a pCMV‐TFG plasmid into osteosarcoma cells. The qRT‐PCR assay validated the overexpression of TFG after transfection with pCMV‐TFG plasmid in the osteosarcoma cells (Figure 6E,F). Meanwhile, WB showed that pCMV‐TFG successfully rescued the decrease in TFG expression caused by CLTC knockdown at the protein level (Figure 6G). Next, the CCK‐8 assay suggested that the up‐regulation of TFG rescued the inhibitory effect of proliferation caused by CLTC knockdown (Figure 6H). The same conclusion could be drawn from the colony formation assay (Figure 6I, Figure S20A). Moreover, the overexpression of TFG also inhibited the increase in apoptotic rate caused by down‐regulation of CLTC (Figure 6J, Figure S20B). Finally, overexpression of TFG inhibited the activity of ER stress induced by CLTC knockdown and ameliorated the inhibitory effects of the TGF‐β and AKT/mTOR signaling pathways caused by CLTC knockdown (Figure 6K). These findings collectively suggested that TFG functioned as an oncogenic effector of CLTC.

### 2.7 TFG correlates with CLTC expression in osteosarcoma samples

**FIGURE 7:**
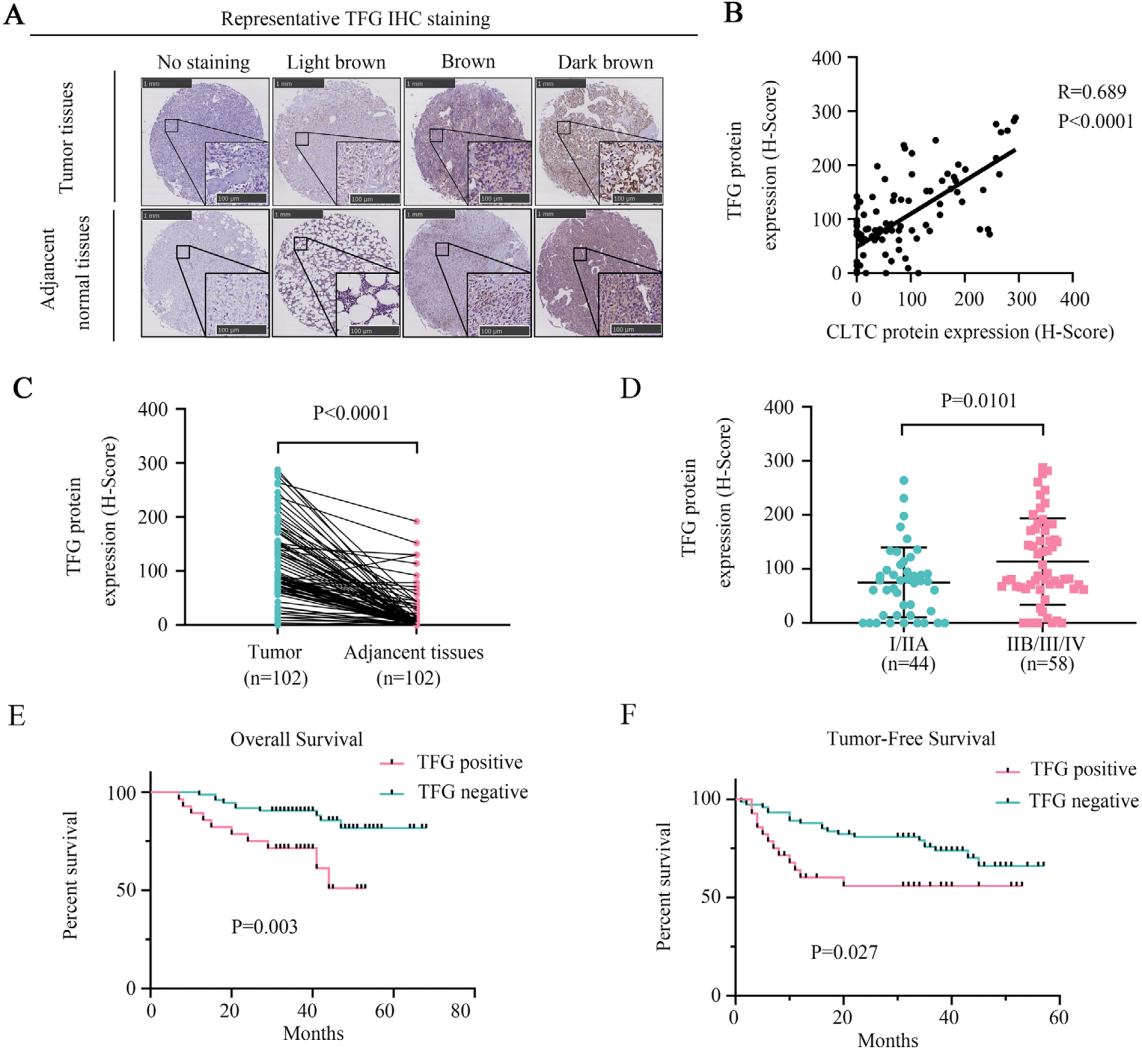
TFG correlated with the expression of clathrin heavy chain (CLTC) in the osteosarcoma samples. (A) Osteosarcoma TMA was used to examine the clinical relevance of TFG expression levels on the patients’ outcomes. A representative IHC‐stained image is shown. (B) Pearson's correlation assay detected the correlation between the TFG and CLTC expression. (C) The IHC result showed the expression of TFG in the osteosarcoma tissues and matched adjacent normal tissues. (D) IHC analysis to quantify the expression of TFG in patients with clinical early stage (I/IIA) versus advanced stage (IIB/III/IV) tumor. (E) Kaplan–Meier analysis was used to determine the overall survival of TFG‐positive and TFG‐negative patients. (F) Kaplan–Meier analysis was used to determine the tumor‐free survival of the TFG‐positive and TFG‐negative patients

To investigate the correlation between TFG and CLTC in clinical samples, we used osteosarcoma TMA to demonstrate their relationship. Representative results of IHC staining of TFG are shown in Figure 7A. A strong positive Pearson correlation was observed between CLTC and TFG (*r* = 0.689; *p* < .0001) (Figure 7B). The IHC results (H score) showed that TFG was significantly more highly expressed in the osteosarcoma tissues than in the matched normal tissues (Figure 7C). Based on the clinical analysis, the expression of TFG was positively related to the AJCC/TNM stage of patients with osteosarcoma (Figure 7D). The Kaplan–Meier survival assay indicated that the tumor‐free survival and overall survival of patients with positive TFG expression were significantly shorter than those of the patients with negative TFG expression (Figure 7E,F).

**FIGURE 8:**
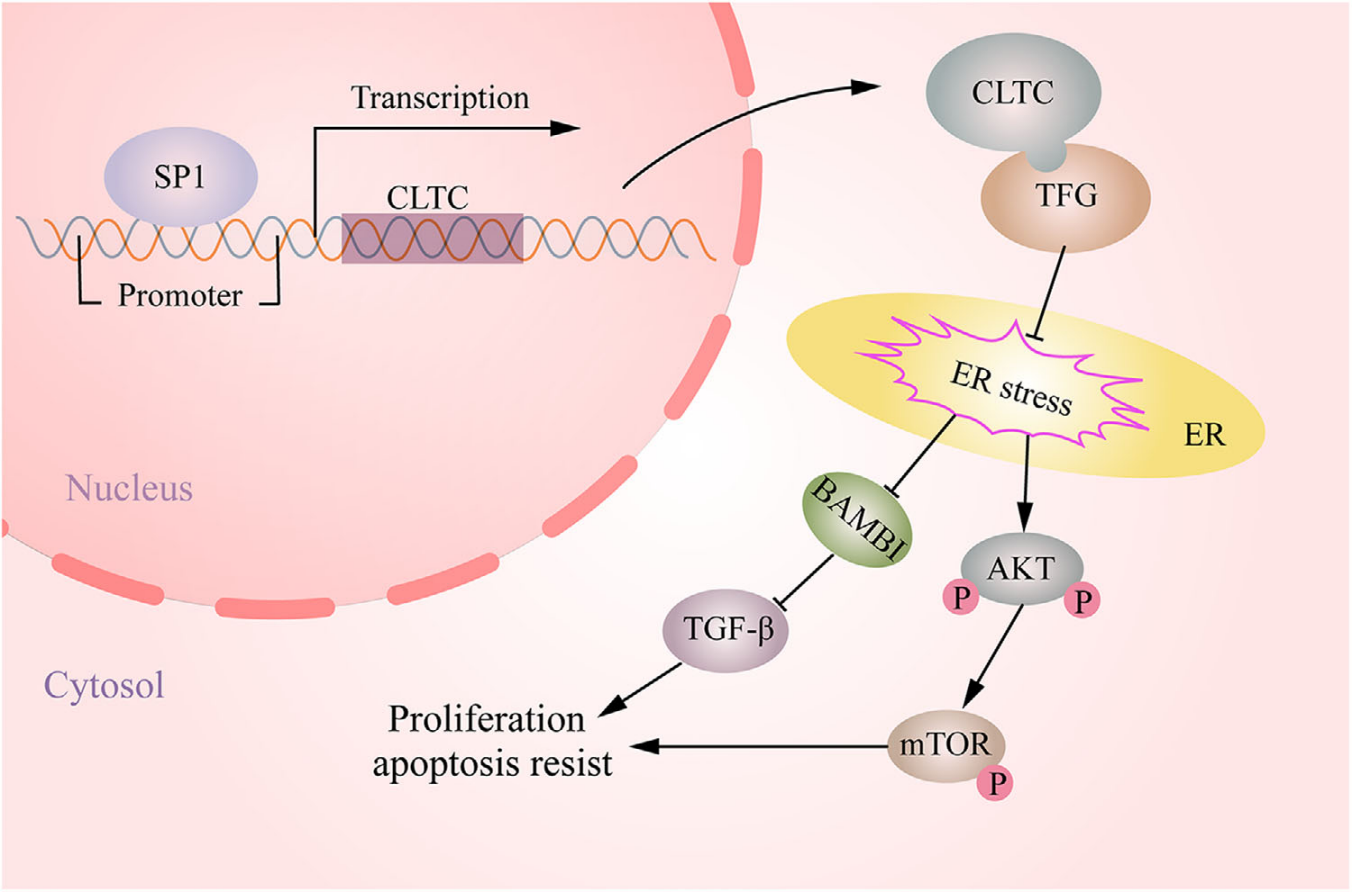
The integrated model showed that SP1 promotes the expression of clathrin heavy chain (CLTC) in osteosarcoma. Moreover, CLTC exerts its oncogenic effects by interacting with TFG, thereby activating the TGF‐β and AKT/mTOR signaling pathways in an ER stress‐mediated manner

In conclusion, this study demonstrated that the expression of CLTC was an independent prognostic factor for patients with osteosarcoma. SP1 promotes the transcriptional activity of *CLTC*. CLTC exerts its oncogenic effects by interacting with TFG, thereby activating the TGF‐β and AKT/mTOR signaling pathways in an ER stress‐mediated manner (Figure 8).

## 3 DISCUSSION

Osteosarcoma is one of the most common malignant bone tumors and is characterized by phenotypic heterogeneity, a high mutational rate, and a varying number of somatic copy number alterations.^26^ After both chemotherapy and surgery, the 5‐year survival rate of patients with osteosarcoma was maintained at 70%. However, the treatment regimen of patients with osteosarcoma remains unchanged in 40 years.^4^ With a variety of genome sequencing methods used to improve the understanding of osteosarcoma and explore the new theory of osteosarcoma treatment, many sequence mutations have been detected in osteosarcoma.^27^ However, all of these mutations failed to provide the key information to improve the prognosis of patients with osteosarcoma.^28^ Therefore, there is an urgent need to further study the molecular pathogenesis of osteosarcoma.

In this study, *CLTC* was selected and considered as a therapeutic target in osteosarcoma. In previous studies, CLTC–ALK fusion was detected in lung cancer,^29^ anaplastic large‐cell lymphomas,^9^ and inflammatory myofibroblastic tumor^30^ and was correlated with the tumor progression. By transcriptome analysis, CLTC–TFE3 fusion was found to exist in kidney cancers and affects many downstream cancer‐related pathways.^31^ Numerous evidences support the concept that CLTC fusion proteins are involved in oncogenesis and tumor progression; however, the role of CLTC itself has not been studied in depth. The role of CLTC in osteosarcoma has also not been explored. In this study, CLTC was confirmed to be an independent prognostic factor for overall survival and tumor‐free survival of patients with osteosarcoma. Down‐regulation of CLTC inhibited cell proliferation, promoted apoptosis, and blocked the cell cycle transition in osteosarcoma. This promising new datum prompted us to analyze the potential mechanism for CLTC deregulation in osteosarcoma.

Transcription factors (TFs) are binding partners that bind to specific gene promoters and mediate chromatin and gene transcription. Deregulation of this complex system can result in aberrant gene expression in cancer.^32^, ^33^ SP1 knockdown osteosarcoma cells showed lower mRNA and protein levels of CLTC than that in the control cells in our study. Therefore, SP1 may regulate the expression of CLTC and mediate the proliferation of osteosarcoma. In previous study, SP1 was confirmed to regulate the transcriptional activation of abundant genes. For example, SP1 overexpresses the long noncoding RNA TUG1, and promotes tumor growth in hepatocellular carcinoma.^34^ In gastric cancer, SP1 binds to the *MTA2* gene promoter, up‐regulates *MTA2* gene expression, and facilitates gastric cancer cell invasion and migration.^35^ The high GC content sequence in the region of *CLTC* promoter, the potential SP1 binding region, also indicates that *CLTC* may be a target gene of SP1. This observation is supported by our findings. Further studies confirmed that SP1 binds to the *CLTC* promoter at the −320 to −314‐nt and +167 to +173‐nt loci and promotes the transcriptional activity of *CLTC* in osteosarcoma. Thus, we first revealed the linkage between transcription factor SP1 and *CLTC* expression in osteosarcoma. Although the intrinsic mechanisms leading to abnormal gene expression are very complex, our findings partly account for the high expression of CLTC in osteosarcoma.

The transcriptional activity of *CLTC* has been shown to be enhanced by SP1 in our study. However, how CLTC plays a pro‐tumor function in osteosarcoma remains unclear. Based on the RNA‐seq analysis findings, the TGF‐β and AKT/mTOR signaling pathways were suggested to play potential roles downstream of CLTC. Interestingly, a previous study has confirmed that CLTC is playing a role in the non‐canonical signaling of TGF‐β. Down‐regulation of CLTC attenuates TGF‐β‐mediated anti‐apoptotic effect in hepatocytes.^36^ Our study also confirmed these findings in osteosarcoma. Moreover, WB also confirmed that CLTC knockdown could inhibit the activation of AKT/mTOR signaling in osteosarcoma. Numerous studies have identified that TGF‐β and AKT/mTOR signaling pathways were extremely relevant to the biological process of osteosarcoma. Ma et al. reveal that TGF‐β is highly expressed and promotes tumor proliferation and metastasis in osteosarcoma.^37^ Saito et al. highlight that TGF‐β promotes the interaction between osteosarcoma cells and platelets, which facilitates tumor progress in osteosarcoma.^38^ Previous studies confirm that hyperactivation of the AKT/mTOR signaling pathway contributes to tumorigenesis, and leads to aberrant tumor growth and invasion, especially in osteosarcoma.^39^, ^40^, ^41^ As these two signaling pathways are suggested to have a pro‐tumorigenic effect in osteosarcoma,^42^, ^43^ our results partly explain the molecular mechanism underlying the pro‐tumor effect of CLTC. Taken together, these findings revealed that CLTC played a pro‐tumor function by the TGF‐β and AKT/mTOR signaling pathways.

Many phenomena in biology, such as proliferation, apoptosis, cell cycle control, and signal transduction, are regulated by the interaction of proteins.^44^, ^45^ Herein, we identified TFG as an interacting protein of CLTC and as an oncogene in osteosarcoma. TFG, a kinase fusion partner plays an important role in regulating protein secretion and links ER sequestration of kinases to oncogenesis.^46^ In the case of TFG knockout, the formation of COPII‐coated transport vehicles was inhibited. This results in a slowdown of secretory cargo transport from ER to the Golgi apparatus and initiates an ER stress response.^16^ The ER stress triggered by prolonged protein overload can affect many signaling pathways.^47^ Upon ER stress, ER stress‐mediated relocalization of GRP78 can block TGF‐β signaling.^48^ ER stress induces phosphorylation of rictor to prevent activation of AKT signaling.^49^ Our results confirm that both CLTC and TFG can regulate protein trafficking from ER to Golgi. Meanwhile, down‐regulation of both CLTC and TFG was found to activate ER stress in osteosarcoma. Therefore, we infer that the interaction of CLTC and TFG may affect the TGF‐β and AKT/mTOR signaling pathways in an ER stress‐mediated manner. In a further study, TFG was overexpressed in osteosarcoma cells by pCMV‐TFG vectors. We observed that overexpression of TFG rescued the inhibition of proliferation and promotion of apoptosis induced by CLTC knockdown. Importantly, the overexpression of TFG inhibited the activation of ER stress caused by CLTC knockdown and rescued the TGF‐β and AKT/mTOR signaling pathways inhibited by the down‐regulation of CLTC. Based on these data, it is reasonable to infer that CLTC‐mediated oncogenic effects occur in a TFG‐dependent manner.

What are the therapeutic implications of our findings? The deregulation of CLTC and TFG is emerging as a unique target for osteosarcoma. On a mechanistic level, knockdown of CLTC and TFG could block the activation of ER stress and suppress their pro‐tumor function. Small‐molecule CLTC inhibitors, such as AK306, are discovered,^42^ and the function of TFG is further understood. Thus, inhibition of CLTC or its interaction with TFG provides a valuable approach for developing effective pharmacological strategies for the treatment of osteosarcoma. As pro‐tumorigenic TGF‐β and AKT/mTOR signaling pathways were driven by CLTC in osteosarcoma, our study also encourages the development of TGF‐β inhibitors and AKT inhibitors for osteosarcoma therapy. In addition, CLTC and TFG can be used as postoperative pathological indicators to predict the prognosis of patients with osteosarcoma. In conclusion, our study provides mechanistic insights into the oncogenic effect of CLTC, and understanding of the SP1/CLTC/TFG axis serves a novel therapeutic strategy for patients with osteosarcoma.

## 4 METHODS

### 4.1 Cell cultures

U2OS, Saos‐2, hFOB1.19, and MNNG/HOS were obtained from the Shanghai Institute for Biological Sciences. All these cell lines were cultured following the protocol of American Type Culture Collection (ATCC). None of the cells had mycoplasma contamination.

### 4.2 Transfection

A Lipofectamine 2000 transfection kit (Invitrogen, USA) was utilized for transfection. 2.0 × 10^4^ Cells were seeded in six‐well plates. Plasmid DNA or si‐RNA was diluted in 200‐uL DMEM in sterile Eppendorf tubes. The liposome suspension was also diluted in 200‐uL DMEM in another sterile Eppendorf tube. Both tubes were maintained for 5 min, and then the two tubes were mixed. The mixed solution was incubated for 20 min and then added to the well. The transfected cells were cultured for an additional 48 h and then collected for the experiment. The si‐RNA was purchased from Guangzhou RUIBO Biosciences (Guangzhou, China).

### 4.3 CCK‐8 and colony formation assay

CCK‐8 assay (Dojindo, Japan) was performed following the manufacturer's instruction. The optical density at 450 nm (OD450) was used to reflect the number of cells. In colony formation assay, 1000 cells were seeded in six‐well plates and cultured at 37°C in the incubator under 5% carbon dioxide. After phosphate buffer saline (PBS) rinsing, the cell colonies were fixed with methanol. Finally, the cell colonies were stained and then assessed under a light microscope.

### 4.4 Cell migration assay

Transwell filter chambers were used in the cell migration assay. Fifty thousand cells were added into the upper chamber per well. Meanwhile, 800 uL of DMEM containing 10% fetal bovine serum (FBS) was added into the lower chamber. After indicated times (MNNG/HOS at 37°C for 14 h, U2OS for 18 h, and Saos‐2 for 48 h), the cells were fixed. After staining with crystal violet, the fixed cells were assessed under microscope.

### 4.5 RNA extraction and PCR experiment

The total RNA was extracted by TRIzol reagent. The DNA impurities were removed with a ribonuclease‐free DNase kit (Qiagen, Australia). PrimeScript RT Reagent Kit (TaKaRa, China) was used to synthesize complementary DNA (cDNA) following the manufacturer's instruction. The qRT‐PCR experiments were conducted by PCR Master Mix (TaKaRa, China). Sequences of the primers used in this study were included in the Supporting Files.

### 4.6 WB

The cells or tissues were lysed by a Protein Extraction Reagent Kit (Thermo Fisher, USA) according to the manufacturer's instruction. Protein quantification was performed with bicinchoninic acid. After gel electrophoresis, the proteins were transferred to a polyvinylidene difluoride (PVDF) membrane. Then, 5% skimmed milk was used to block. Finally, proteins were detected by specific antibodies.

### 4.7 Immunofluorescence

After 15 min of infiltration using 4% formaldehyde, the cells were incubated with 0.3% Triton X‐100 for 10 min. After PBS rinsing, the wells were infiltrated with blocking solution for 30 min, and then incubated with CLTC and TFG antibody at 4°C for more than 8 h. After that the secondary antibody was added to the wells and incubated for 1 h in dark conditions. DAPI (YEASEN, China) was used to stain the nucleus. Images were observed and recorded using a confocal laser scanning microscope (Leica TCS‐SP5, Germany).

### 4.8 Xenograft model experiment

Four‐week‐old BALB/c nude mice were used for tumorigenicity assays. All of the nude mice were kept in specific pathogen‐free conditions. The nude mice were subcutaneously injected in the right flank with 1 × 10^6^ MNNG/HOS cells with sh‐CLTC or sh‐NC (eight per group). The length and width of tumors were measured at the indicated times. Thirty days after injection, the tumor‐bearing mice were sacrificed. Then, the tumors were removed from the mice for further study. For inhibition experiments, all tumor‐bearing mice started dosing when average tumor volume reached 30–50 mm^3^. Tumor‐bearing mice were randomized into four groups (*n* = 8). Mice fed for (1) saline, (2) Palbociclib, (3) Perifosine, (4) co‐delivery Palbociclib + Perifosine, with a Palbociclib dose of 150 mg/kg/day and a Perifosine dose of 36 mg/kg/day. Tumor sizes were measured every 2 days. After 2 weeks, all mice were sacrificed and the tumor tissues were collected for further study. The volume of tumor was calculated as (length × width^2^) × 0.5. All experiments were conducted after approval by the Animal Care and Use Committee of Shanghai Cancer Institute.

### 4.9 Co‐IP and MS analysis

Cells were collected and lysed with RIPA buffer (Beyotime, China). The cell lysis was precleared by incubation with the magnetic beads for 2 h. Then, the cell lysis was incubated with primary antibodies at 4°C for more than 8 h. After that, 30 μl magnetic beads were incubated with the cell lysis for 2 h, and then PBS/0.1% Triton rinses were performed. The magnetic beads were collected using a magnetic rack, then boiled with 2× SDS‐loading buffer. The sample was stained by a Silver Staining Kit (Beyotime, China) after electrophoresis, and then split with trypsin. Finally, samples were collected and identified by mass spectrometer.

### 4.10 Cell apoptosis and cell cycle analysis

After 48 h, the transfected cells were collected and measured via an Apoptosis Detection Kit (BD, USA). The data were analyzed by FlowJo software version 8.6.3 (FlowJo, USA). Cell cycle distribution was measured using a Cell Cycle Detection Kit (Beyotime, China). Then, ModFit software (BD Biosciences, USA) was used to analyze the results.

### 4.11 Clinical samples and IHC

Eight pairs of osteosarcoma tissues and matched normal tissues were collected from the patients who had surgical resection performed at Shanghai No. 6 Hospital in 2007. Then these eight pairs of samples were used for RNA‐seq analysis. Meanwhile, we selected 102 patients with confirmed osteosarcoma who had surgical resections performed at Shanghai No. 6 Hospital from 2014 to 2017, and for whom postoperative pathological tissues were available for IHC. Tissue microarray (TMA) containing these 102 cases of osteosarcoma tissues and matched normal tissues were prepared by the Department of Orthopedics. All patients agreed to analyze their tumors through a protocol approved by the Review Committee of Shanghai No. 6 Hospital. Then, the relevant clinical information was collected.

Slides were dewaxed in xylene and rehydrated using 100%, 95%, and 75% gradient alcohol. Then, the slides were boiled for 20 min using EDTA solution. After cooling for 10 min, PBS rinses were performed. After primary antibody incubation for 1 h at 37°C, the slides were incubated for 30 min with secondary antibodies (Invitrogen, USA). Diaminobenzidine (DAB) was then used as a chromogen to develop for 5 min, followed by hematoxylin staining for 2 min. After PBS rinsing, the slides were blued in lithium carbonate before mounting with cover slips and glycerin. The ratio of the stained cells and the degree of staining were used as evaluation criteria. The ratio of positive stained cells was scored as: <5% (0), 5–25% (1), 25–50% (2), 50–75% (3), and >75% (4). The intensity of staining was scored as no staining (0), light brown (1), brown (2), and dark brown (3). The final IHC scores were obtained using both the traditional scoring method and the H score method. In traditional scoring method, IHC scores were calculated as the product of intensity (0–3) and the ratio of positively stained cells (0–4), yielding a range from 0 to 12. Positive expression (high expression) was defined as IHC scores ≥8, and negative expression (low expression) was defined as IHC scores <8. The H score of IHC was determined according to the formula: [(% of light brown) × 1] + [(% of brown) × 2] + [(% of dark brown) × 3], yielding a range from 0 to 300.

### 4.12 Luciferase assay and ChIP

Primers used to amplify the CLTC promoter are included in the Supporting File. The promoter fragments of CLTC, mutant type, or wild type promoter sequence were inserted into pGL3‐Basic vector. pRL‐TK Renilla luciferase plasmids were used for internal control. After 48‐h transfection, dual luciferase assays were conducted by a dual luciferase assay kit (Promega, USA) following the manufacturer's instruction. ChIP assays were performed by the Pierce Agarose ChIP Kit (Thermo Fisher, USA) according to the manufacturer's protocol. Osteosarcoma cells were incubated with formaldehyde for 10 min to cross‐link the protein and DNA. Cell lysates were then sonicated on ice for 30 min and immunoprecipitated with SP1 antibody or IgG. The precipitated DNA was collected and then analyzed by PCR assay. The primers for PCR assay are included in the Supporting File.

### 4.13 DNA pull‐down assay

CLTC promoter fragments containing SP1 binding sites or mutant SP1 binding sites were synthesized. The synthesized double‐stranded DNA fragments were purified and biotin‐labeled using the Biotin Random Primer Labeling Kit (Biomart, Beijing, China). The biotin‐labeled DNA fragments were then incubated with streptavidin‐conjugated magnetic beads and binding buffer following the manufacturer's protocol. After preclearing, the nuclear lysate was incubated with the DNA‐probe magnetic bead complex for 2 h. After washing three times with binding buffer, the magnetic beads were boiled with 2× sample buffer and analyzed via WB. Oligo A1 contains −320 ∼ −314 nt SP1 binding site, and oligo A2 contains +167 ∼ +173 nt SP1 binding site.

### 4.14 Statistics

All of the statistical analysis was assessed by SPSS 22.0 software (IBM, USA). The staging of osteosarcoma was based on the AJCC/TNM system of eighth edition. The significance of the difference among groups was estimated using Student's two‐tailed *t*‐test or the χ^2^ test. The correlation between the CLTC and TFG expression was tested by Pearson's correlation assay. The survival curves were calculated by the Kaplan–Meier log‐rank test. The tumor‐free survival time was defined as the time from operation to the appearance of new local lesions. The time from operation to death was defined as the overall survival time. The results were the mean value ± standard deviation of one typical experiment in three independent experiments, and each representative experiment was repeated in triplicate. The *p*‐values less than .05 were considered statistically significant.

## CONFLICT OF INTEREST

The authors declare **that** there is no conflict of interest.

## AUTHOR CONTRIBUTIONS

Cheng Dongdong, Yang Qingcheng, and Li Shijie conceived and designed the experiments. Li Shijie, Pan Zhen, Qin Kang, and Guo Hua performed the experiments. Li Shijie and Pan Zhen analyzed the data. Li Shijie and Cheng Dongdong wrote the manuscript. All the authors read and approved the final manuscript.

## Supporting information

Supporting Information

## Data Availability

All data included in this study are available upon request by contact with the corresponding author.
