# Supplementary material for: Deregulation of CLTC interacts with TFG, facilitating osteosarcoma via the TGF‐beta and AKT/mTOR signaling pathways
Source: Clin Transl Med. 2021 Jun 20;11(6):e377. doi: 10.1002/ctm2.377 (PMC8214859; doi:10.1002/ctm2.377)
Supplement: Supplementary file 1 — Supporting Information [file CTM2-11-e377-s001.pdf]

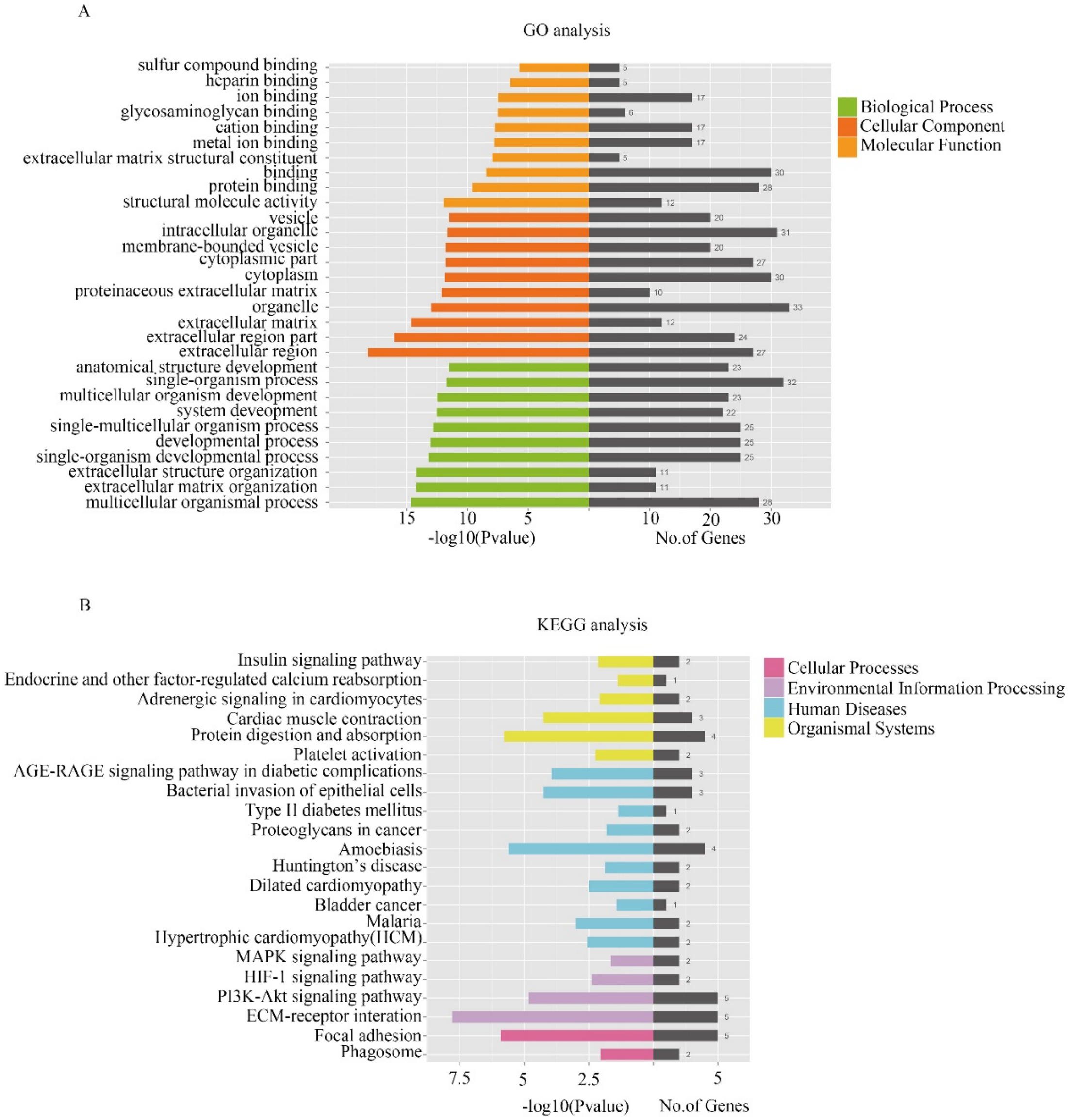

Figure S1. The GO and KEGG analysis of RNA-seq of 8 pairs of osteosarcoma tissues and matched adjacent normal tissues. (A) The GO analysis of RNA-seq of 8 pairs of osteosarcoma tissues and matched adjacent normal tissues. (B) The KEGG analysis of RNA-seq of 8 pairs of osteosarcoma tissues and matched adjacent normal tissues.

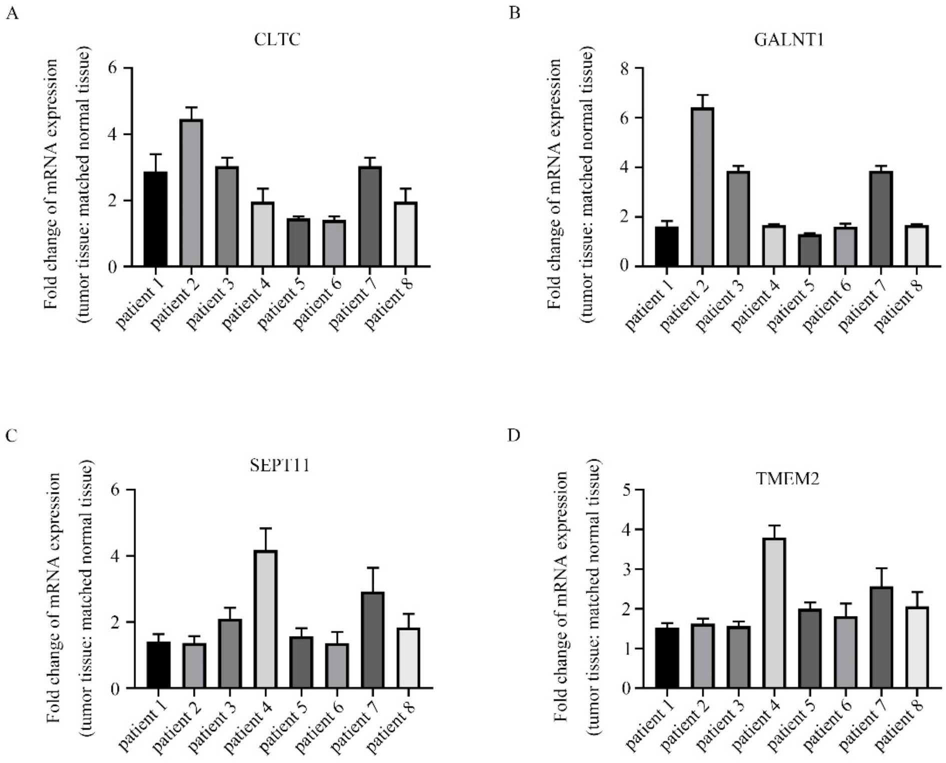

Figure S2. The mRNA expression levels of CLTC, GALNT1, SEPT11 and TMEM2 in the 8 pairs of osteosarcoma tissues and matched adjacent normal tissues.

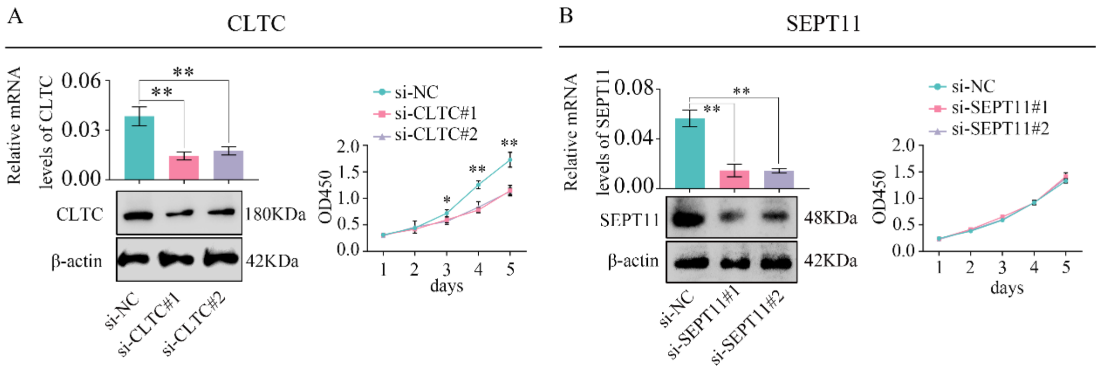

Figure S3. Si-RNA knockdown and CCK-8 assay was performed to search for proliferation-related gene in MNNG/HOS cell line. Levels of mRNA and protein expression were validated after specific siRNAs transfection in MNNG/HOS cells by qRT-PCR and WB, respectively. CCK-8 assays were performed after siRNA transfection.

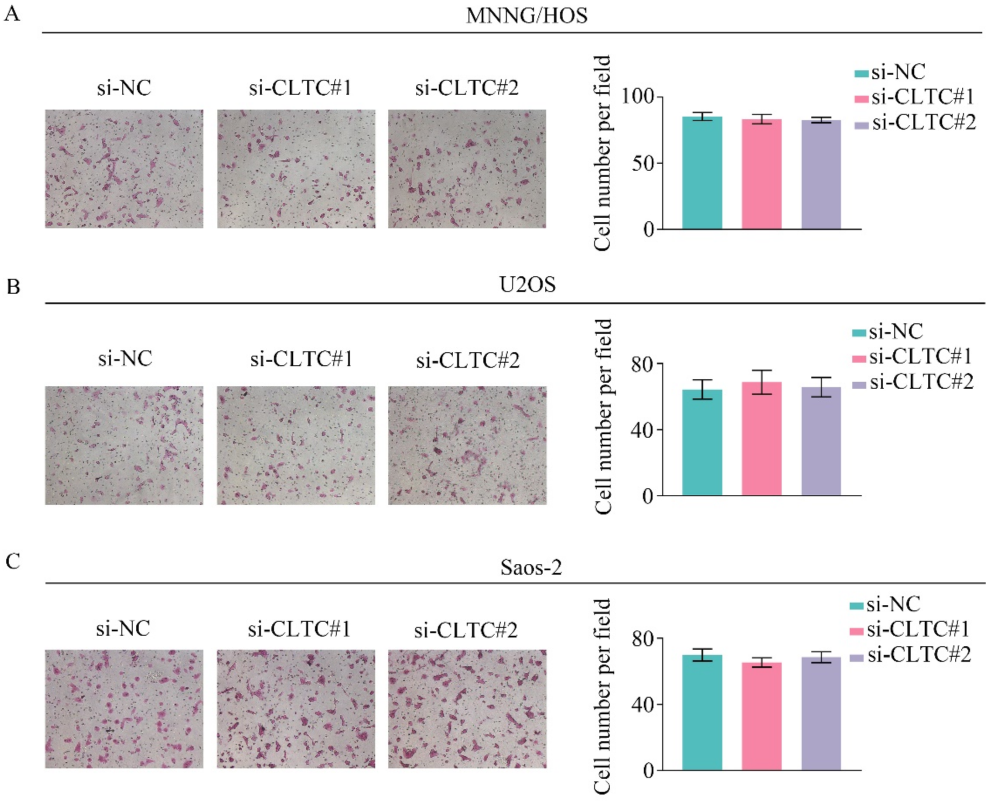

Figure S4. Transwell migration assays in osteosarcoma cells transfected with two independent CLTC siRNAs (magnification, ×200).

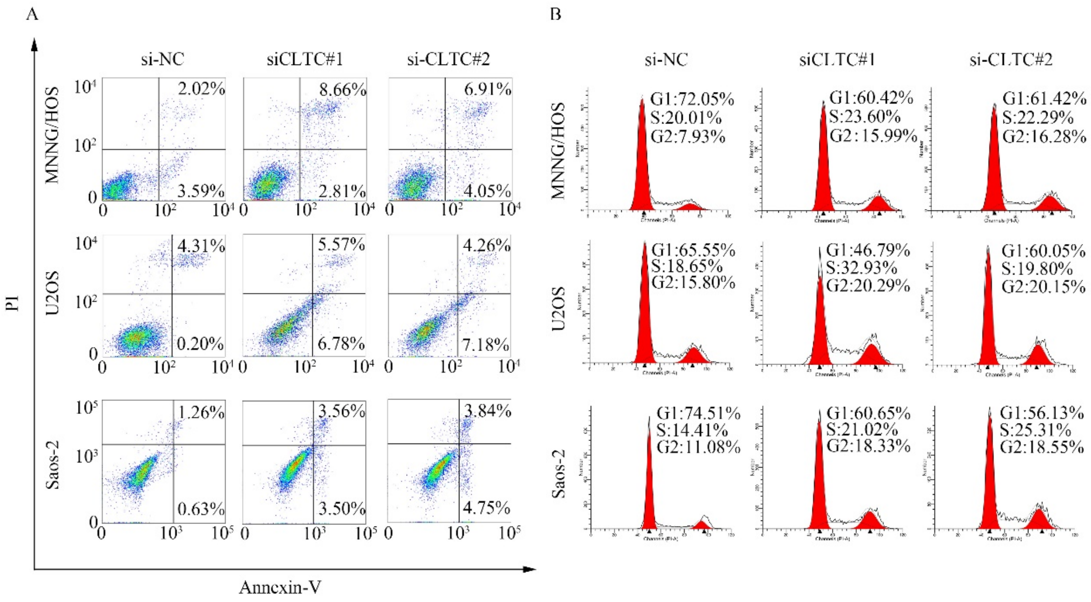

Figure S5. Representative diagram of apoptosis percentages and the cell cycle between CLTC- silence cells and control cells (A) Representative diagram of apoptosis percentages of CLTC-silence cells and control cells confirmed by flow cytometry. (B) Representative diagram of the cell cycle of CLTC-silence cells and control cells confirmed by flow cytometry.

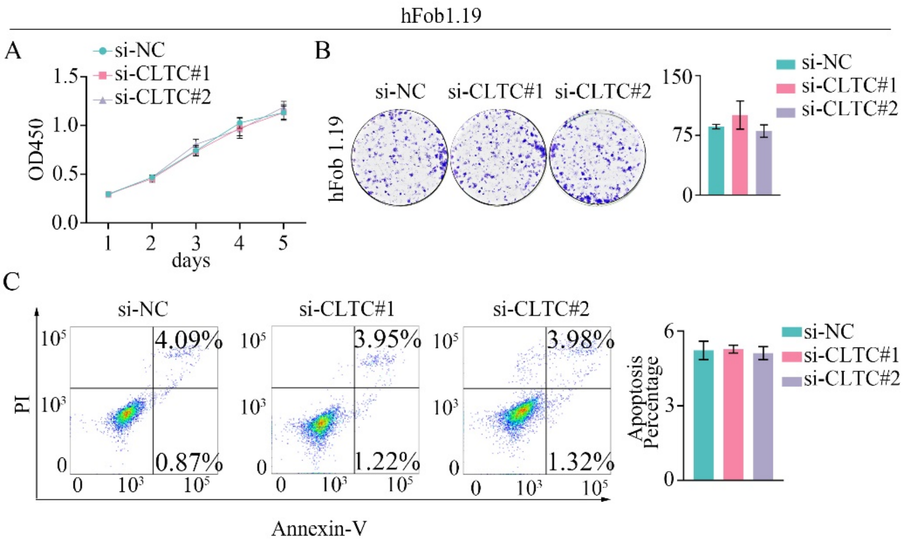

Figure S6. Effects of knockdown of CLTC on osteosarcoma cells. (A) CCK-8 assay was performed in the MNNG/HOS, U2OS, and Saos-2 cells after CLTC knockdown. (B) Colony formation assay was performed in the MNNG/HOS, U2OS, and Saos-2 cells after si-CLTC transfection. (C) The apoptosis percentages of CLTC knockdown cells and control cells confirmed by flow cytometry.

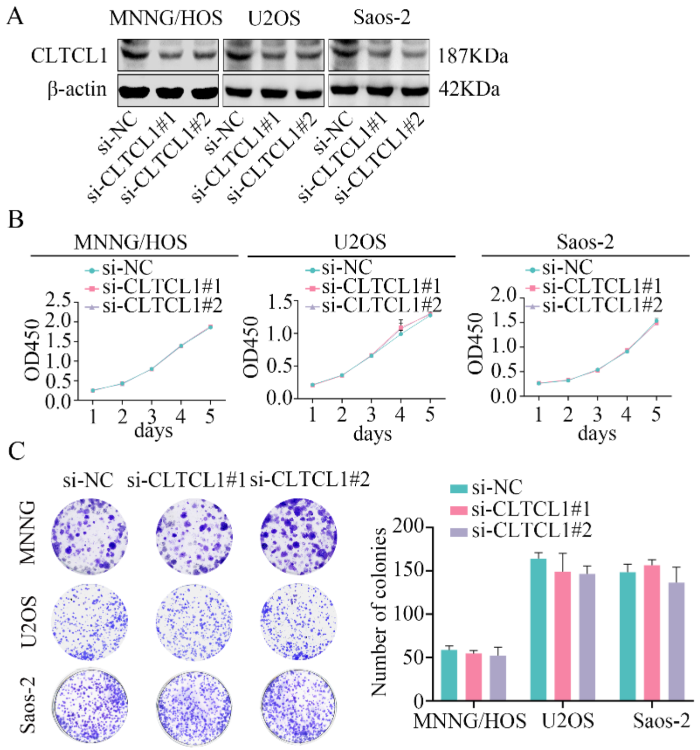

Figure S7. Effects of knockdown of CLTCL1 on osteosarcoma cell proliferation. (A) After transfection of two independent CLTCL1 siRNAs in the MNNG/HOS, U2OS, and Saos-2 cells, the protein levels of CLTCL1 were verified. (B) CCK-8 assay was performed in the MNNG/HOS, U2OS, and Saos-2 cells after CLTCL1 knockdown. (C) Colony formation assay was performed in the MNNG/HOS, U2OS, and Saos-2 cells after si-CLTCL1 transfection.

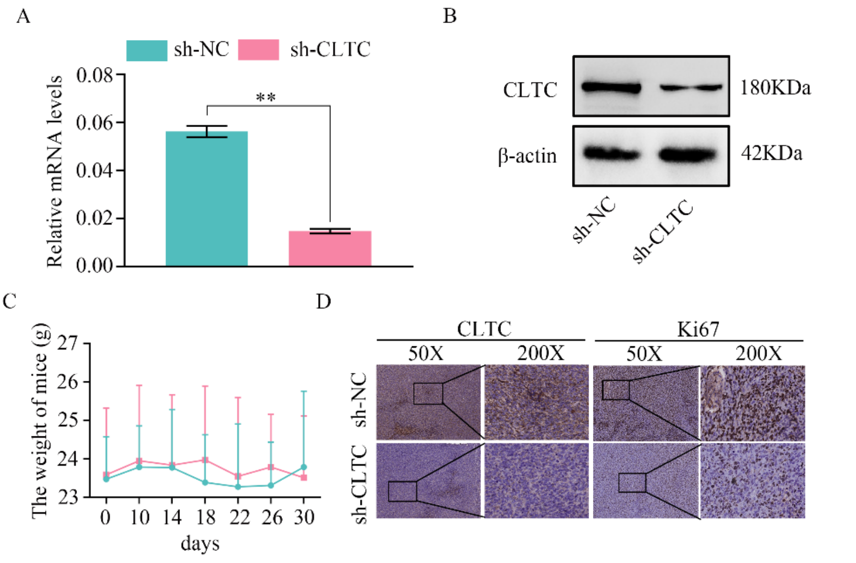

Figure S8. CLTC knockdown inhibited the proliferation of osteosarcoma cells in vivo. The qRT- PCR (A) and WB (B) were used to detect the expression of CLTC in MNNG/HOS cells stably transfected with sh-NC or sh-CLTC. (C) Weights of the xenograft tumor model mice on indicated days. Values represent the mean ± SD. (D) Representative IHC results stained by CLTC and Ki67 antibody in xenograft tumors formed by CLTC-silence and control cells.

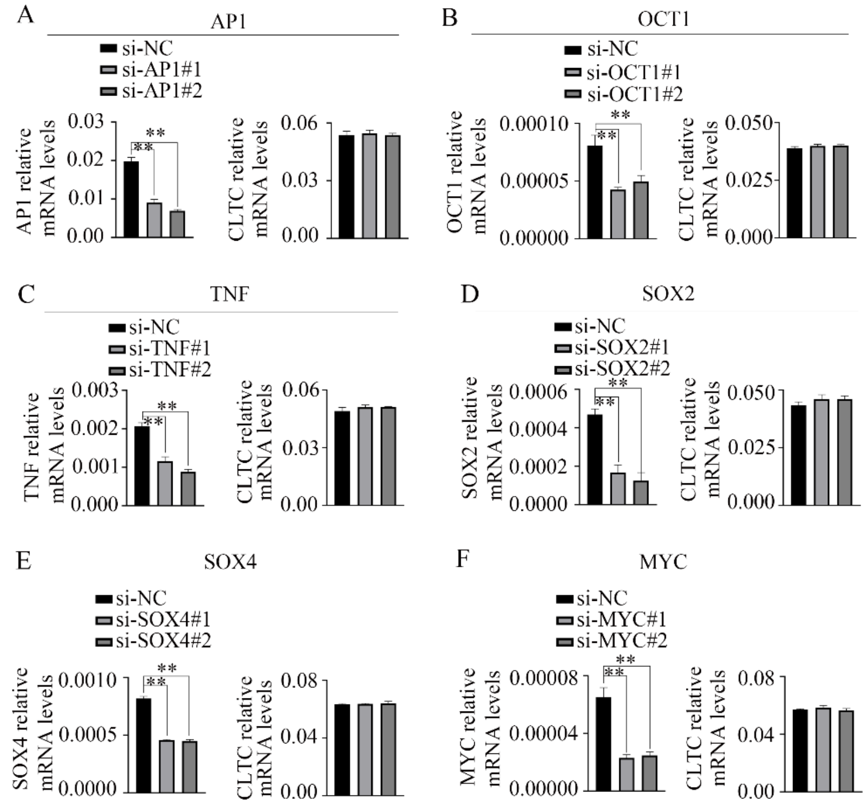

Figure S9. The qRT-PCR was used to detect the expression of CLTC in MNNG / HOS cells after AP1, OCT1, TNF, SOX2, SOX4 and MYC targeted siRNAs transfection.

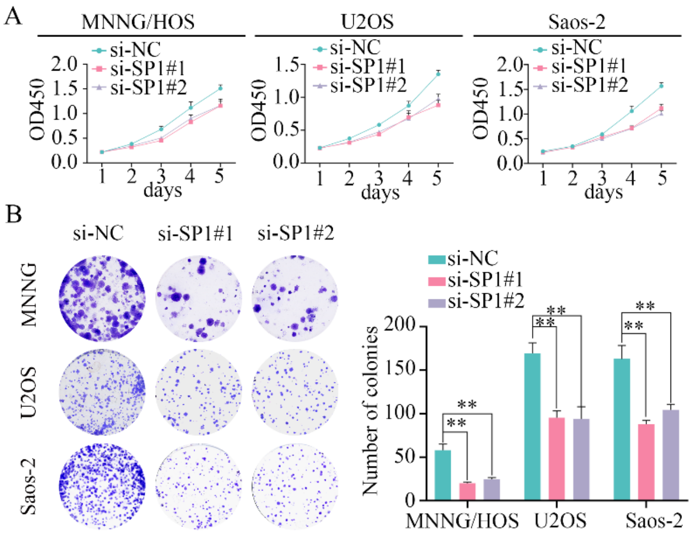

Figure S10. Effects of knockdown of SP1 on osteosarcoma cell proliferation. (A) CCK-8 assay was performed in the MNNG/HOS, U2OS, and Saos-2 cells after SP1 knockdown. (C) Colony formation assay was performed in the MNNG/HOS, U2OS, and Saos-2 cells after si-SP1 transfection.

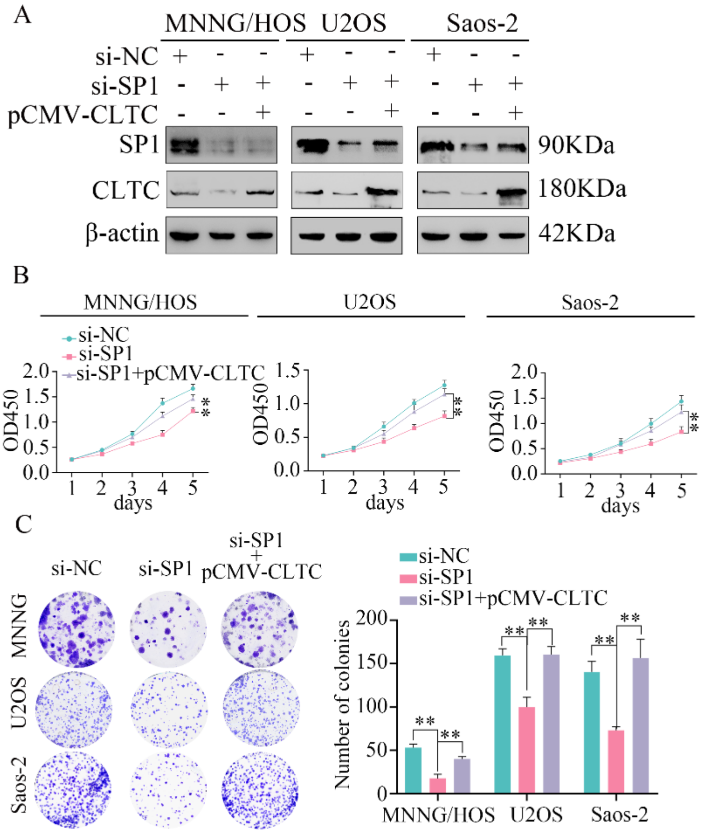

Figure S11. Overexpression of CLTC rescued the tumor-suppressive effect of SP1 knockdown in the osteosarcoma cells. (A) WB showing the SP1 and CLTC protein expression in the osteosarcoma cells transfected with si-NC and si-SP1 and co-transfected with si-SP1 and pCMV-CLTC. (B) CCK-8 assays were used to determine the cell proliferation for the osteosarcoma cells transfected with si-NC and si-SP1 and co-transfected with si-SP1 and pCMV-CLTC. (C) Colony formation assays for the osteosarcoma cells transfected with si-NC and si-SP1 and co-transfected with si-SP1 and pCMV-CLTC.

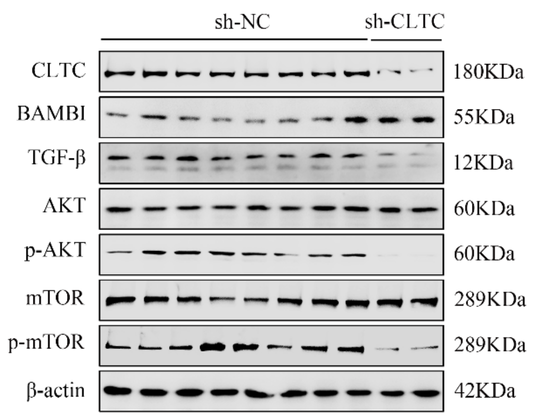

Figure S12. Representative blots show the protein levels of BAMBI, TGF-β, AKT, p-AKT, mTOR, and p-mTOR in the xenograft tumor tissues. β-actin served as the internal control.

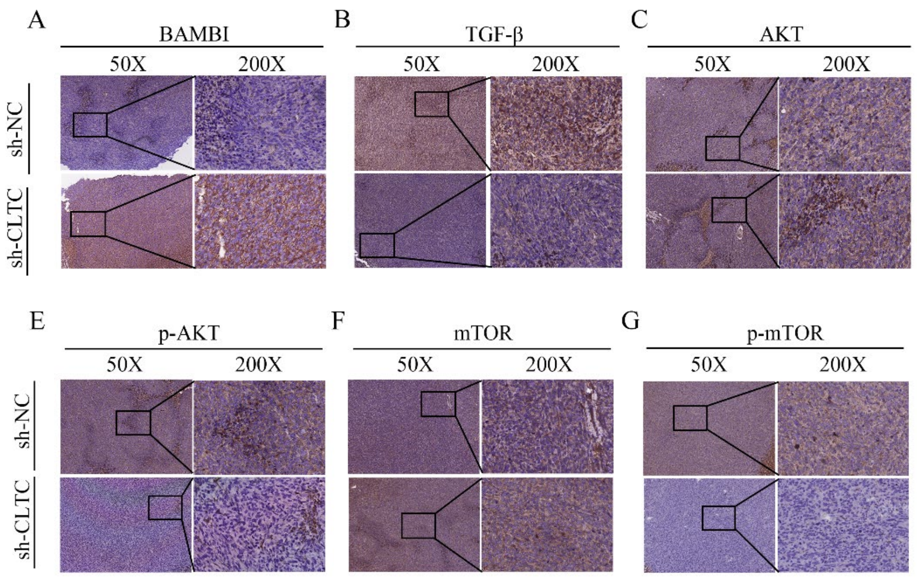

Figure S13. Representative IHC results showed the protein levels of BAMBI, TGF-β, AKT, p-AKT, mTOR and p-mTOR in sh-NC xenograft tumors and sh-CLTC xenograft tumors.

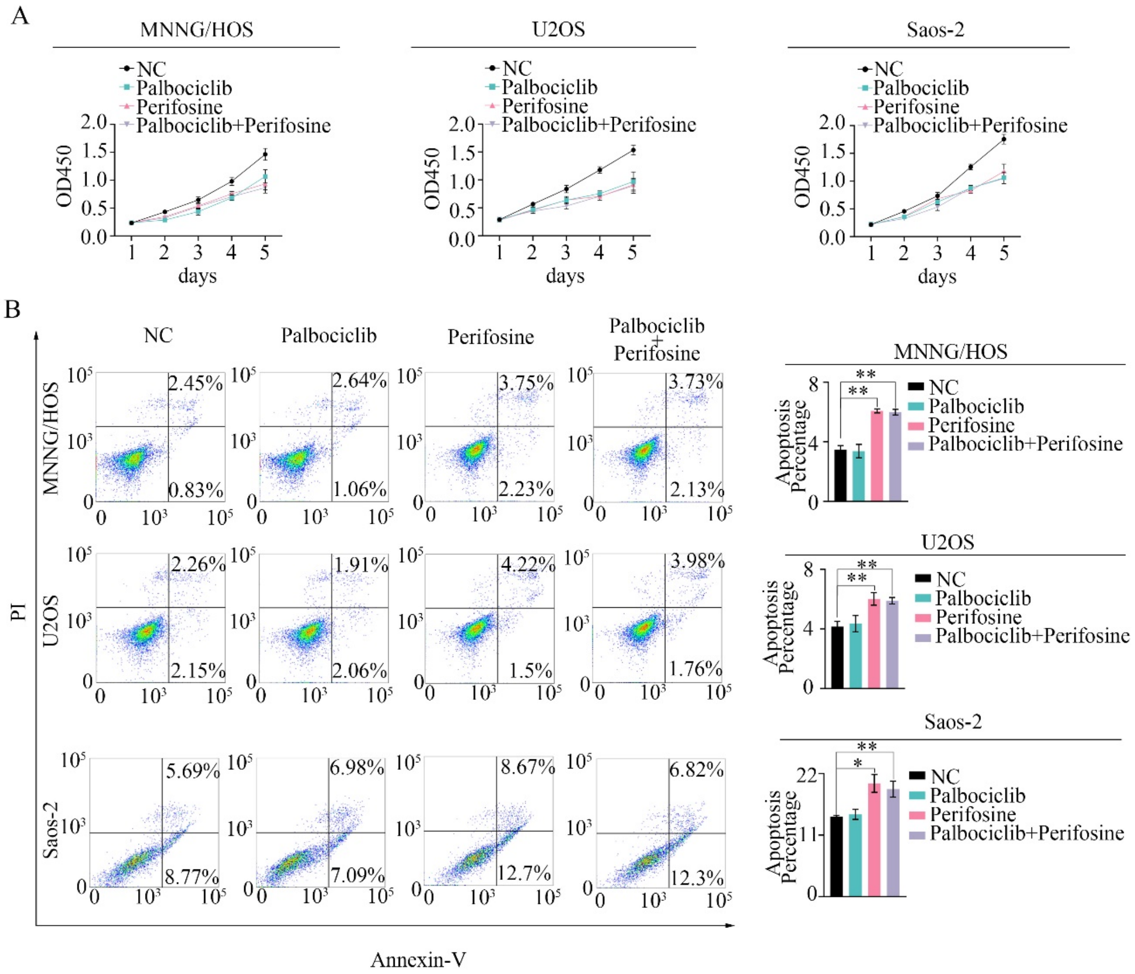

Figure S14. The effects of Palbociclib and Perifosine in osteosarcoma cells in vitro. (A) CCK-8 assay was performed after Palbociclib (1uM), Perifosine (5uM), or a combination of Palbociclib (1uM) and Perifosine (5uM) was treated to osteosarcoma cells. (B) The apoptosis percentages of saline, Palbociclib (1uM), Perifosine (5uM), or a combination of Palbociclib (1uM) and Perifosine (5uM) treated cells were confirmed by flow cytometry.

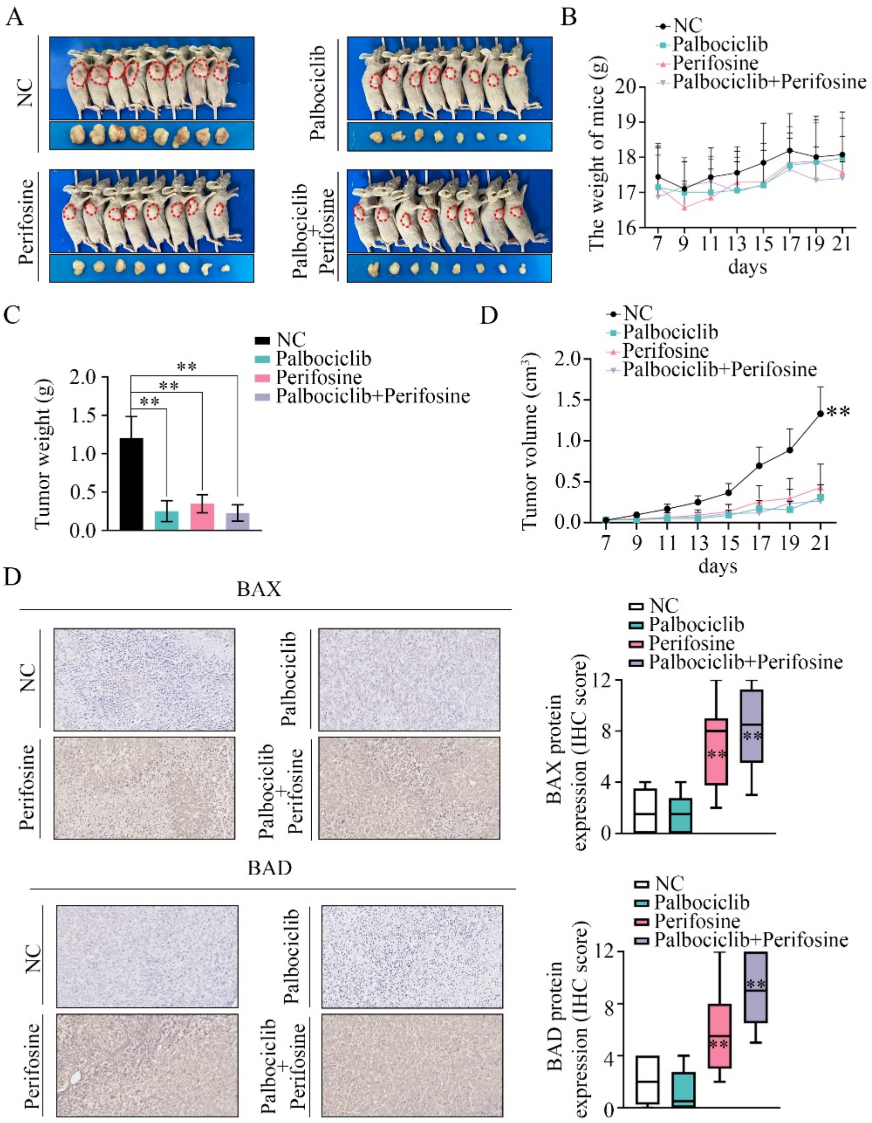

Figure S15. The effects of Palbociclib and Perifosine in osteosarcoma cells in vivo. (A) Saline, Palbociclib, Perifosine, or a combination of Palbociclib and Perifosine were used to treat tumor-bearing mice. (B) Weights of the tumor-bearing mice on indicated days. (C) The weight of the xenograft tumors in the NC, Palbociclib, Perifosine, and a combination of Palbociclib and Perifosine groups. (D) Growth curve demonstrating the tumor volumes on indicated days. (E) IHC results showed the protein levels of BAX and BAD in NC, Palbociclib, Perifosine, and a combination of Palbociclib and Perifosine treated xenograft tumors.

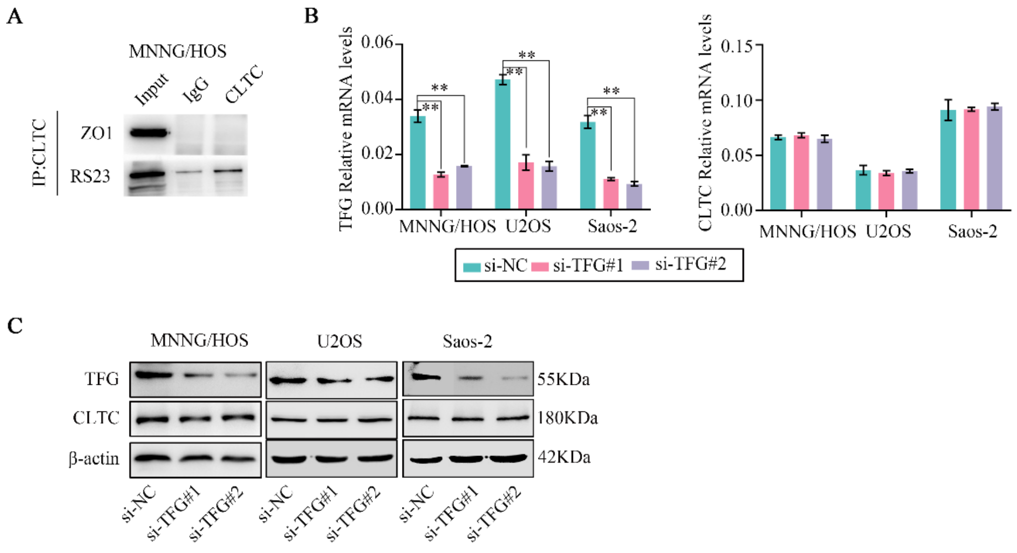

Figure S16. Co-IP-WB was performed to confirm the interacting proteins of CLTC in MNNG/HOS cell line. (A) Cell lysates from MNNG/HOS cell lines were immunoprecipitated with an anti-CLTC antibody followed by immunoblotting (IB) with anti-ZO1 and anti-RS23 antibodies. IgG was used as a negative control. (B) The qRT-PCR was used to detect the expression of TFG and CLTC in osteosarcoma cells transfected with si-NC and si-TFG#1 and si-TFG#2. (C) After the down-regulation of the TFG expression, there were no significant changes in the CLTC expression in the protein levels.

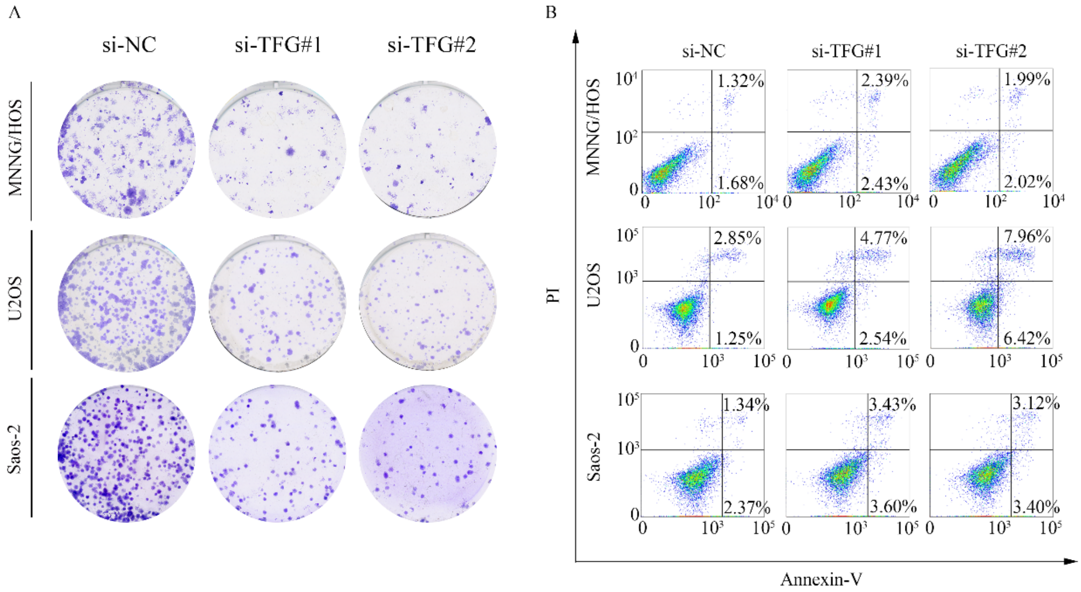

Figure S17. Down-regulation of TFG inhibited the proliferation and promote apoptosis of osteosarcoma cells. (A)Representative diagram of clone forming assay of TFG knockdown cells and control cells. (B) Representative diagram of the apoptosis percentages of TFG knockdown cells and control cells confirmed by flow cytometry.

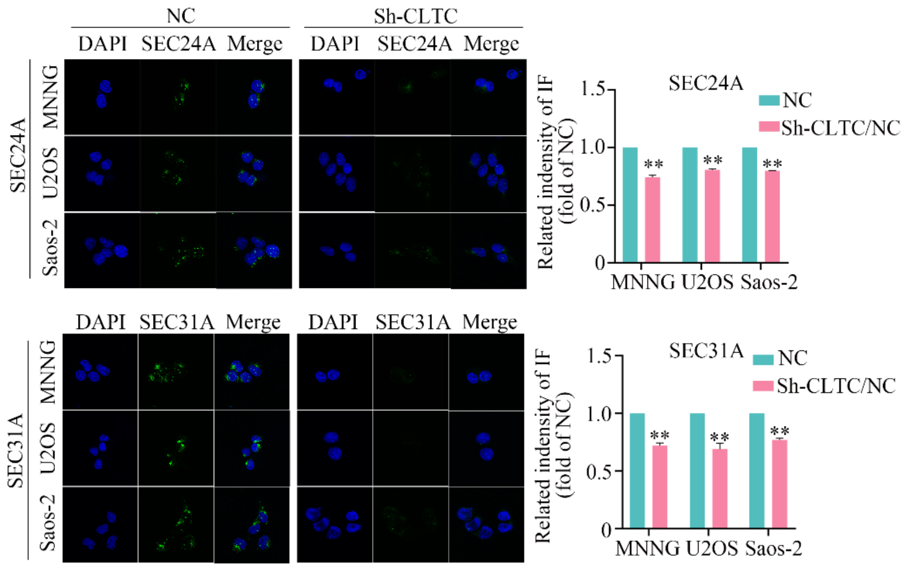

Figure S18. Immunofluorescence images show the protein levels of SEC24A and SEC31A in osteosarcoma cells following CLTC knockdown.

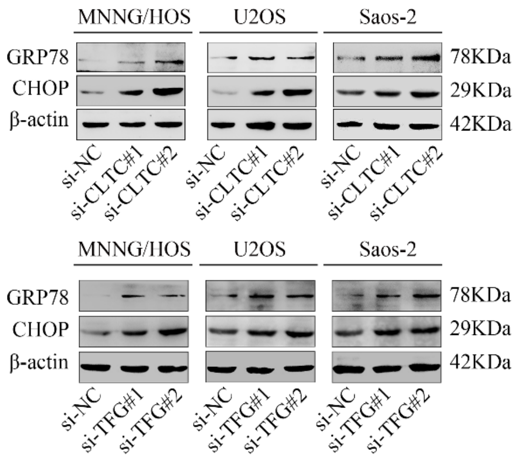

Figure S19. Representative blots of GRP78 and CHOP in the MNNG/HOS, U2OS, and Saos-2 cells after CLTC or TFG knockdown.

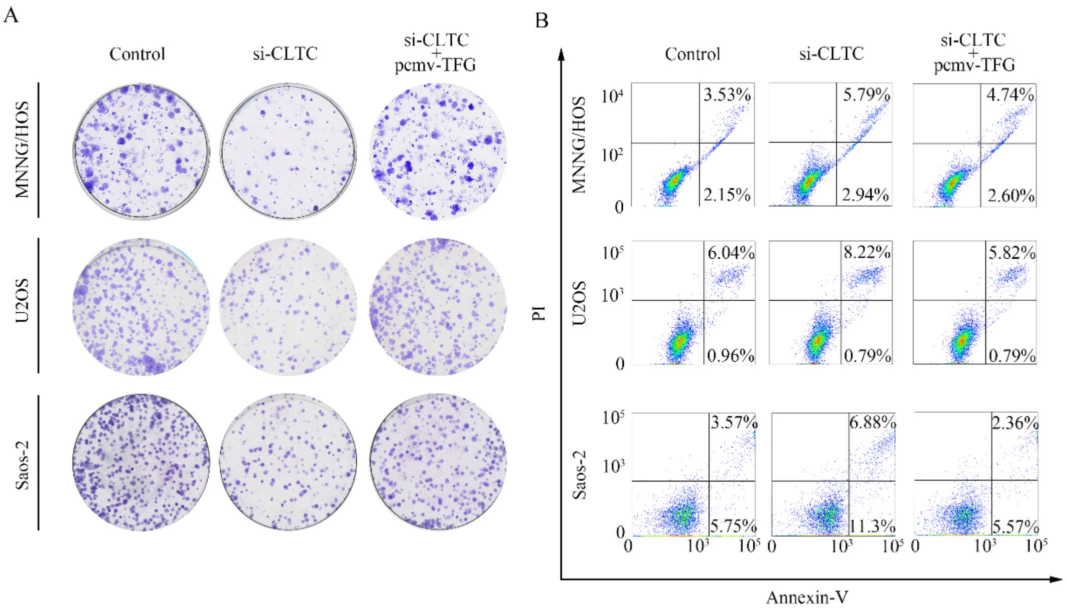

Figure S20. Overexpression of TFG rescued the tumor-suppressive effect of CLTC knockdown. (A) Representative diagram of a clone forming assay of osteosarcoma cells transfected with si-NC and si-CLTC and co-transfected with si-CLTC and pCMV-TFG. (D) Representative diagram of apoptosis percentages of osteosarcoma cells transfected with si-NC and si-CLTC and co-transfected with si-CLTC and pCMV-TFG.

Supporting Figures and Tables

Quantification of immunoblots:

Quantification of immunoblots in Figure 1E

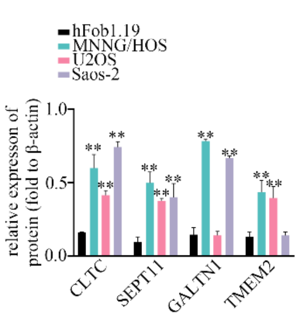

Quantification of immunoblots in Figure 2A,B,C

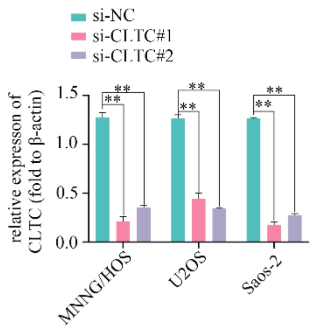

Quantification of immunoblots in Figure 2K

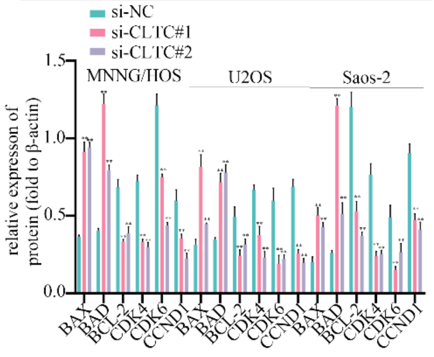

Quantification of immunoblots in Figure 3B

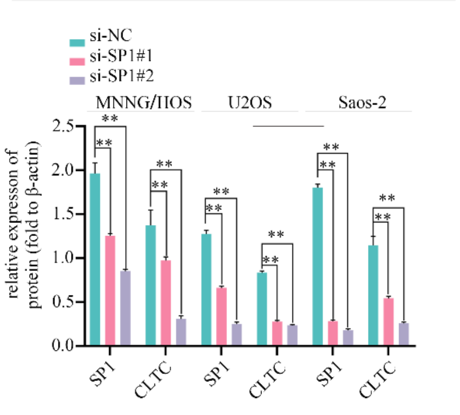

Quantification of immunoblots in Figure 4K

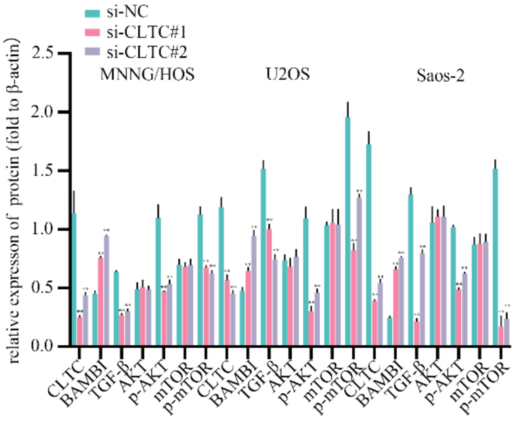

Quantification of immunoblots in Figure 4L

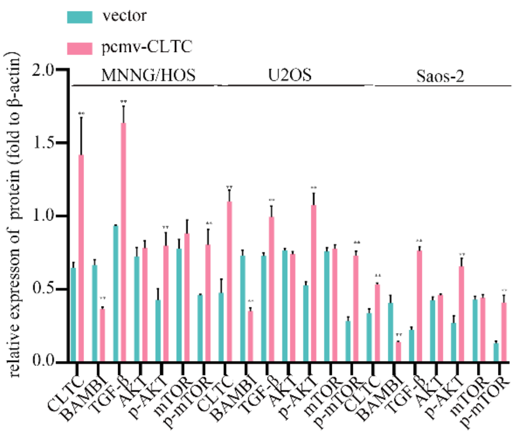

Quantification of immunoblots in Figure 5G

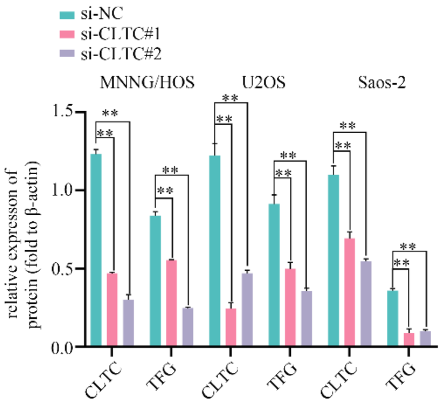

Quantification of immunoblots in Figure 6A

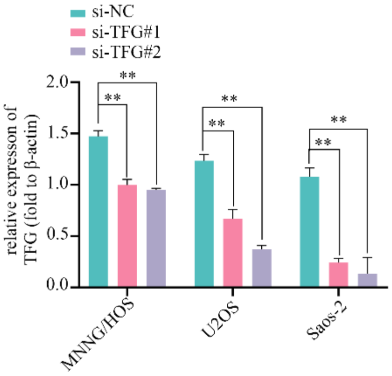

Quantification of immunoblots in Figure 6G

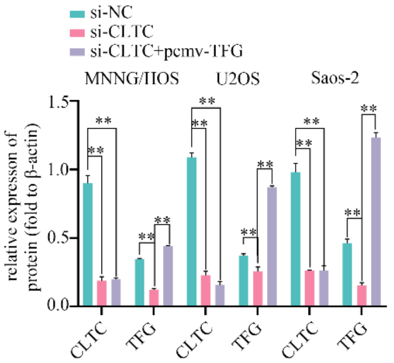

Quantification of immunoblots in Figure 6K

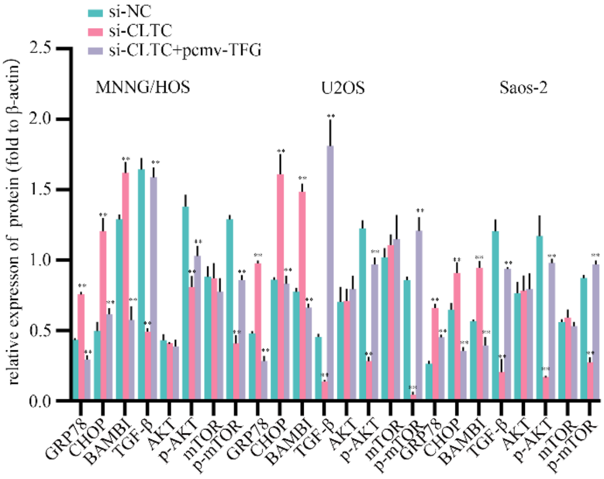

Quantification of immunoblots in Figure S3

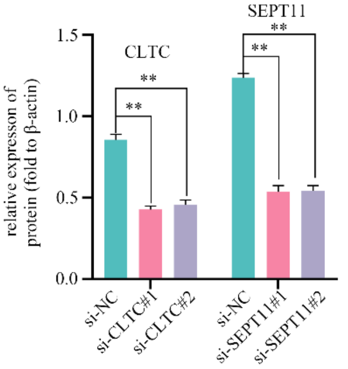

Quantification of immunoblots in Figure S6A

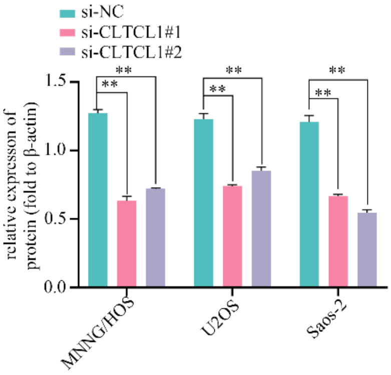

Quantification of immunoblots in Figure S7B

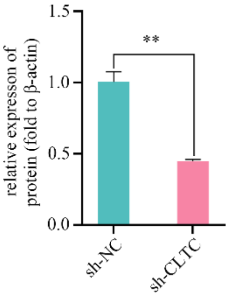

Quantification of immunoblots in Figure S10

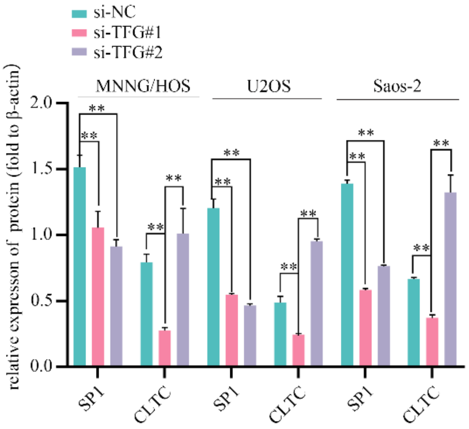

Quantification of immunoblots in Figure S11

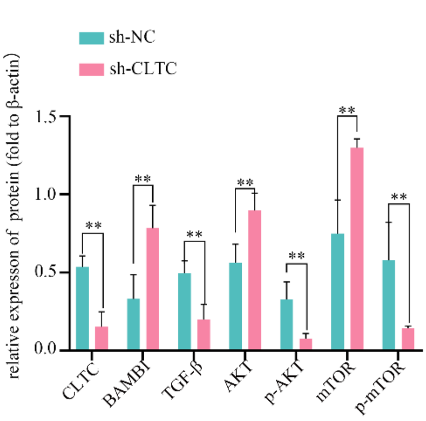

Quantification of immunoblots in Figure S13C

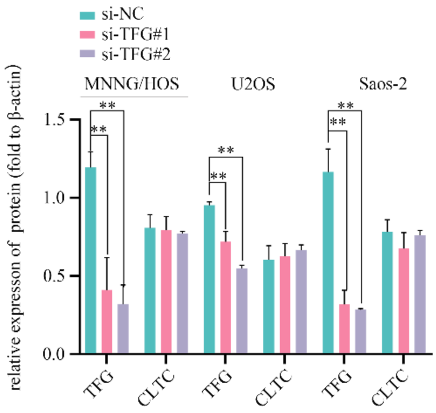

Quantification of immunoblots in Figure S16A

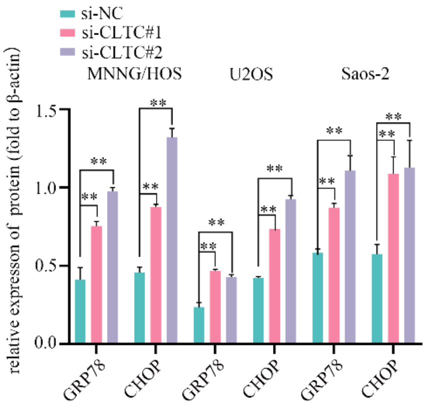

Quantification of immunoblots in Figure S16B

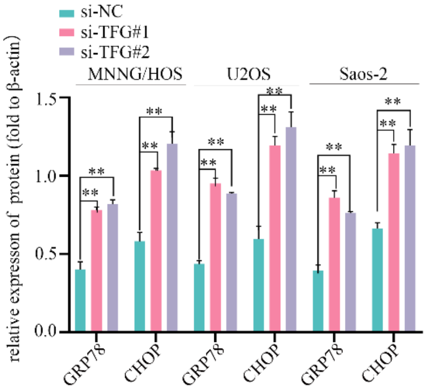

Supplementary Table S1. Primer sequences for PCR reaction

Supplementary Table S1. Primer sequences for PCR reaction		
**Gene**	**Forward primer**	**Reverse primer**
**Primer sets for SYBR-based detection**		
**CLTC**	ACGGTTGCTCTTGTTACGGA	TGCATAGCTCCCACCACACG
**MXRA5**	CTCTCCGTGGTGCTGATCCT	GGTTTCTGACAGGGCCTGTA
**CALU**	ATTATCATGGACCTGCGACA	TCCAAGCCTTTCCTTGCTCT
**GALNT1**	ACTGCAAGGTGGTCCTAGCC	ACGACTGGTTTCCCCATTTC
**TPM4**	CGACGCATCCAGCTCGTTGA	CGCAATGTGCTTGGCCTCTT
**SEPT11**	CTGGCCATGTGGGATTTGAC	ACCGAACACCTGGTTCATTG
**TMEM2**	CAACCTTCGCATTCTCACCT	TGGATCCCAATTCCTGAGAC
**OLFML2B**	GGACAAGCGAGCCCCCAGAT	CCCGCATTGATCCTCTGGCA
**COL5A2**	GCACTCAGAATGGCCAGATG	CACCTCCAGGTGTTTGTGAA
**COL12A1**	CACCAGAGCTGAGGCAGACA	AACTGCCACTCTGTTCTGGG
**SULF1**	GCTCTGGTTTTGGCTGTCCT	GGGCAGCACATGGGTGTAGT
**FN1**	CCTCGAAGAGCAAGAGGCAG	CCACTCGGTAAGTGTTCCCA
**TFG**	TGATCAGGTTTCAGGGCCAC	TGACCTTCAATCTGACCTGCT
**BID**	AAGGAGGAAGCGGGTAGTCG	AACCGTTGTTGACCTCACAGT
**SP1**	CCACCATGAGCGACCAAGAT	TGAAAAGGCACCACCACCAT
**MAPK9**	ACCCTTCGGGATATTGCAGG	TGCAGCACAAACAATCCCTTG
**IL1A**	GCGTTTGAGTCAGCAAAGAAGT	CATGGAGTGGGCCATAGCTT
**BIK**	GAATGCATGGAGGGCAGTGAC	CTGATGTCCTCAGTCTGGTCG
**TAP1**	GCCTTGTTCCGAGAGCTGAT	AATGGCCATCTCCCCAAGAG
**CTH**	CTCACTGTCCACCACGTTCA	CCTGTTTGTACAGTACTTAGCCC
**CASP7**	GTGGGAACGATGGCAGATGA	TTCCGTTTCGAACGCCCATA
**CCND2**	AACTCAAAGAGACCAGCCCG	ACTTAAAGTCGGTGGCACACA
**PDCD4**	ATGTAAACCCTGCAGAAAATGCT	GGATCGCCTATCCAGCAACC
**CASP2**	ACAGTTACCTGCACACCGAG	GGGATCCAATAGCACCTCCAC
**CASP8**	TTCACCTTGTGTCTGAGCTGG	CAGGGGCTGCTCCTTCTTC
**IL2RG**	TGTTTGCATTGGAAGCCGTG	ACTCCAGGCCGAAAAGTTCC
**NGF**	GGCAGACCCGCAACATTACT	CACCACCGACCTCGAAGTC
**PTEN**	TGGATTCGACTTAGACTTGACCT	GGTGGGTTATGGTCTTCAAAAGG
**RIT1**	CGCGAGTGAAGGAAGACGAA	TGCATGGTCATGGCTCACAA
**AKT1**	GCACAAACGAGGGGAGTACA	AAGGTGCGTTCGATGACAGT
**YWHAB**	GTCTCCAGAGATTTGGGCCG	CCATTGTCATTCCCTGACTCCA
**EGFR**	AAATGGGCTGCAAAGCTGTC	GTTTCCCCCTCTGGAGATGC
**PMEPA1**	AGGCGAAAAGTCAAAATGCCT	CAGGCATCCTTCTGAGGACA
**SERPINE1**	ACCGCAACGTGGTTTTCTCA	TTGAATCCCATAGCTGCTTGAAT
**HIPK2**	CCCCGTGTACGAAGGTATGG	GGGATGTTCTTGCTCTGGCT
**KLF10**	CGGGCGCGATTATGCAATTA	TCCATTCTTTCCTCCATTTCTCC
**UBE2D3**	AGTTGCAGCTTTTCACCCGA	AACACTCCACCCTTGCCATC
**SMAD1**	CTTTCCAGCAACCCAACAGC	CAACCGCCTGAACATCTCCT

**FNTA**	ATGGACGACGGGTTTGTGAG	TCCGGAAATGCCACACTGTAT
**SFN**	TGACGACAAGAAGCGCATCAT	GTAGTGGAAGACGGAAAAGTTCA
**GSK3B**	TTGGACTAAGGTCTTCCGACCC	TGAATCCGAGCATGAGGAGG
**SMAD3**	AGCGCACTGACCATAAGAGC	CATCCAGGGACTCAAACGTGG
**NOG**	CCATGCCGAGCGAGATCAAA	TCGGAAATGATGGGGTACTGG
**SMURF**	ACTGGAGCTGATCATAGGCG	TTGAAGCCTTGGAGCGGG
**β-actin**	TATAAAACCCGGCGGCGCA	TCATCCATGGCGAACTGGTG
**Primer sets for chromatic immunoprecipitation assay**		
**CLTC-SP1-P1-Mut**	TGAGTCACGGAGAGGaaGGGAGGCTGCGCCC	GGGCGCAGCCTCCCttCCTCTCCGTGACTCA
**SP1-P2-Mut**	GGTGAGGCGACTGGaaGGAGTTCTGTCGC	GCGACAGAACTCCttCCAGTCGCCTCACC

Supplementary Table S2. List of antibodies				
**Antibody**	**Source**	**Catalog number**	**Reagent brand**	**Applications**
CLTC	mouse monoclonal	66487-1-Ig	proteintech	WB, IF, IHC
CLTC	Rabbit Polyclonal	ab21679	abcam	IP
TFG	mouse monoclonal	66916-1-Ig	proteintech	WB, IHC
TFG	Rabbit monoclonal	ab156866	abcam	IP
SP1	Rabbit Polyclonal	21962-1-AP	proteintech	WB
BAX	Rabbit Polyclonal	50599-2-Ig	proteintech	WB
p-BAD	Rabbit Polyclonal	#4366	CST	WB
BCL-2	Rabbit Polyclonal	12789-1-AP	proteintech	WB
CDK4	Rabbit Polyclonal	11026-1-AP	proteintech	WB
CDK6	Rabbit Polyclonal	14052-1-AP	proteintech	WB
CCND1	mouse monoclonal	60186-1-Ig	proteintech	WB
ZO1	mouse monoclonal	66452-1-Ig	proteintech	WB
RS23	mouse monoclonal	sc-100837	SANTA CRUZ	WB
GALNT1	Rabbit Polyclonal	ab109918	abcam	WB
SEPT11	Rabbit Polyclonal	14672-1-AP	proteintech	WB
TMEM2	Rabbit Polyclonal	ab98348	abcam	WB
BAMBI	Rabbit Polyclonal	16100-1-AP	proteintech	WB, IHC
TGF-beta	Rabbit Polyclonal	#3711	CST	WB
TGF-beta	mouse monoclonal	MAB1835	NOVUS	IHC
AKT	Rabbit Polyclonal	#4685	CST	WB,IHC
p-AKT	Rabbit Polyclonal	#4060	CST	WB,IHC
m-TOR	Rabbit Polyclonal	#2983	CST	WB,IHC
p-mTOR	Rabbit monoclonal	ab109268	abcam	WB,IHC
beta-actin	Mouse Monoclonal	HRP-60008	proteintech	WB
Ki67	Rabbit Polyclonal	27309-1-AP	proteintech	IHC

Supplementary Table S3. shRNA/siRNA target sequence for gene knockdown

Name	Type	Target sequence
sh-CLTC	sh-RNA	CCTGCGGTCTGGAGTCAAC
si-CLTC#1	si-RNA	CCTGCGGTCTGGAGTCAAC
si-CLTC#2	si-RNA	GCTAAGAGCTGCAGAACAA
si-TFG#1	si-RNA	CCAGGACCTTCCACCAATA
si-TFG#2	si-RNA	GGATCTAAGTGGGAAGCTA
si-SP1#1	si-RNA	GCAACATCATTGCTGCTAT
si-SP1#2	si-RNA	GCCAATAGCTACTCAACTA

Supplementary Table S4. The enriched biological processes and signaling pathways for CLTC down-regulation.

Gene Ontology (Biological Process)

GOBPID	Term	Pvalue	Corrected P-Value
GO:0050808	synapse organization	4.26E-06	0.009695173
GO:0044699	single-organism process	8.20E-06	0.009695173
GO:0032501	multicellular organismal process	1.83E-05	0.014419372
GO:0044707	single-multicellular organism process	3.17E-05	0.018723068
GO:0000904	cell morphogenesis involved in differentiation	0.000138	0.043286146
GO:0048731	system development	0.000153	0.043286146
GO:0051239	regulation of multicellular organismal process	0.000225	0.043286146
GO:0050803	regulation of synapse structure or activity	0.00026	0.043286146
GO:0007275	multicellular organism development	0.000267	0.043286146
GO:0044763	single-organism cellular process	0.000267	0.043286146
GO:0022414	reproductive process	0.000282	0.043286146
GO:0000003	reproduction	0.000287	0.043286146
GO:0099536	synaptic signaling	0.00031	0.043286146
GO:0098916	anterograde trans-synaptic signaling	0.00031	0.043286146
GO:0099537	trans-synaptic signaling	0.00031	0.043286146
GO:0007268	chemical synaptic transmission	0.00031	0.043286146
GO:0048667	cell morphogenesis involved in neuron differentiation	0.000361	0.043286146
GO:0050807	regulation of synapse organization	0.000365	0.043286146
GO:0009987	cellular process	0.000374	0.043286146

KEGG (Pathway Enrichment)

ID	Term	P-Value	Corrected P-Value
hsa04921	Oxytocin signaling pathway	0.052204	0.530448824
hsa04350	TGF-beta signaling pathway	0.074154	0.530448824
hsa04310	Wnt signaling pathway	0.1746	0.57933921
hsa04024	cAMP signaling pathway	0.281252	0.57933921
hsa04662	B cell receptor signaling pathway	0.320547	0.57933921
hsa04010	MAPK signaling pathway	0.387079	0.57933921
hsa04066	HIF-1 signaling pathway	0.4192	0.57933921
hsa04668	TNF signaling pathway	0.440085	0.58735816
hsa04068	FoxO signaling pathway	0.506179	0.635117391
hsa04150	mTOR signaling pathway	0.555283	0.655234277
hsa04390	Hippo signaling pathway	0.555283	0.655234277
hsa04630	Jak-STAT signaling pathway	0.564505	0.659520236
hsa05200	Pathways in cancer	0.616428	0.667325773
hsa04151	PI3K-Akt signaling pathway	0.834272	0.834272483

Supplementary Table S6. List of possible CLTC-interacting proteins identified in MNNG/HOS.

Gene name	score	Coverage	Proteins	Unique Peptides
CLH1	4074.24	47.76	2	69
NONO	184.3	22.29	1	7
HS71A	328.19	16.85	2	6
TFG	84.97	19.5	1	6
CLCA	110.57	13.71	1	5
FLNA	81.24	2.38	3	4
PLAK	39.97	6.31	1	4
MPRIP	39.31	3.8	1	4
ACTC	1144.93	42.97	4	3
RA1L2	140.1	13.13	4	3
GBB2	133.14	17.65	4	3
SRSF6	109.69	7.27	3	3
SP16H	106.99	3.72	1	3
CLCB	67.66	11.35	1	3
GNAS2	51.93	10.91	9	3
HBA	44.65	23.94	2	3
SEM7A	41.07	3.3	1	3
RL35A	40.18	25.45	1	3
ZO2	31.24	2.1	1	3
RS23	21.57	20.98	1	3
GBB1	146.96	15.59	2	2
H2AZ	69.62	12.5	15	2
DX39A	61.21	4.68	2	2
LDHA	60.04	5.72	1	2
MOES	58.77	3.29	3	2
CH60	52.15	3.66	1	2
TMOD3	49.6	5.68	1	2
PHB	48.65	8.09	1	2
TNS2	47.82	1.63	1	2
H3C	47.31	11.85	5	2
ODO2	46.38	4.19	1	2
EF1G	45.27	5.95	1	2
1433Z	43.5	8.16	1	2
ECHA	42.93	3.93	1	2
ZO1	41.43	1.54	1	2
AGRIN	41.1	1.02	1	2
DDX1	40.93	2.57	1	2
XRCC6	40.87	3.45	1	2
LEG8	39.35	5.99	1	2
IF2B2	38.2	5.01	2	2

ANXA2	33.9	7.37	2	2
AP2M1	33.33	3.68	1	2
NEXN	31.2	2.81	1	2
NP1L1	24.3	7.16	1	2
TOP1M	23.37	2.83	2	2

Supplementary Table S6. List of possible CLTC-interacting proteins identified In U2OS.

Gene name	score	Coverage	Proteins	Unique Peptides
K2C4	570.44	30.9	6	13
TBA1B	570.1	35.25	9	11
K1C13	501.52	29.48	4	10
K2C6A	437.72	25.89	5	7
RS2	146.56	26.28	1	7
HNRH1	514.82	24.05	3	6
ANXA1	325.44	16.47	1	4
SNX9	89.32	7.23	1	4
RS23	125.27	23.08	1	3
UBP2L	102.78	2.94	1	3
RS11	99.74	24.68	1	3
IGHA1	44.29	7.93	3	3
TBB5	344.38	25.45	6	2
K2C3	240.87	10.51	2	2
CRNN	121.72	7.88	1	2
RL31	74.12	18.4	1	2
S10A9	74.01	24.56	1	2
RL17	68.9	13.59	1	2
HNRH3	64.74	8.38	1	2
HNRPR	52.77	3.32	2	2
RS9	50.93	8.76	1	2
AMY1	50.52	4.11	3	2
TFG	48.85	4.5	1	2
HSPB1	46.16	12.68	1	2
AXA2L	42.17	7.37	2	2
ZO1	38.51	1.77	1	2
HS90B	36.22	3.31	5	2
RS26	35.55	20.87	2	2
CD109	22.1	1.87	1	2

Supplementary Table S7 **Clinical information for all patients**

Patients	Gender	Age	Location	Operation type	Tumor necrosis rate (%)	Recurrence
	female	14	Distal femur	Amputation	< 90	No
2	female	15	Proximal femur	Limb salvage	< 90	No
3	female	23	Proximal tibia	Limb salvage	< 90	No
4	male	19	Distal femur	Limb salvage	< 90	No
5	female	24	Proximal femur	Limb salvage	< 90	No
6	female	33	Proximal humerus	Amputation	≥90	Yes
7	female	12	Distal femur	Amputation	< 90	Yes
8	female	14	Proximal tibia	Limb salvage	< 90	No
9	female	12	Distal radius	Amputation	< 90	Yes
10	female	13	Distal femur	Limb salvage	< 90	No
11	male	8	Distal femur	Limb salvage	< 90	No
12	male	13	Proximal tibia	Amputation	< 90	Yes
13	male	7	Pelvis	Limb salvage	< 90	No
14	male	33	Proximal tibia	Limb salvage	< 90	No
15	male	18	Distal radius	Limb salvage	< 90	Yes
16	female	17	Distal femur	Limb salvage	< 90	Yes
17	female	13	Distal femur	Limb salvage	≥90	No
18	female	22	Distal femur	Limb salvage	≥90	No
19	female	16	Distal femur	Limb salvage	< 90	No
20	female	38	Distal femur	Amputation	≥90	No
21	female	19	Distal radius	Limb salvage	≥90	No
22	female	35	Proximal tibia	Amputation	≥90	Yes
23	female	14	Distal femur	Limb salvage	< 90	No
24	male	27	Proximal femur	Limb salvage	< 90	Yes
25	female	31	Proximal tibia	Limb salvage	< 90	No
26	female	12	Distal femur	Limb salvage	≥90	No
27	female	35	Distal femur	Limb salvage	< 90	No
28	male	16	Pelvis	Limb salvage	< 90	Yes
29	male	19	Distal femur	Limb salvage	< 90	No
30	male	34	Proximal tibia	Limb salvage	< 90	Yes
31	female	17	Distal femur	Amputation	< 90	No
32	female	14	Proximal tibia	Limb salvage	< 90	No
33	female	33	Distal femur	Limb salvage	< 90	No
34	female	11	Proximal tibia	Limb salvage	< 90	No
35	female	27	Distal femur	Amputation	< 90	Yes
36	male	34	Distal femur	Limb salvage	< 90	Yes
37	female	10	Proximal humerus	Amputation	< 90	No
38	female	15	Distal femur	Limb salvage	≥90	No
39	male	31	Proximal humerus	Limb salvage	< 90	No
40	female	11	Proximal tibia	Limb salvage	< 90	No

Patients	Metastasis	AJCC/TNM stage	Tumor-Free Survival (Month)	Overall Survival (Month)	CLTC expression	TFG expression
1	Yes	IIA	37	64	Negative	Negative
2	Yes	IIA	35	65	Negative	Negative
3	Yes	IV	34	67	Negative	Negative
4	Yes	III	43	64	Negative	Negative
5	No	IIA	57	57	Negative	Negative
6	No	III	20	44	Positive	Positive
7	No	III	10	20	Positive	Positive
8	Yes	III	56	56	Negative	Negative
9	No	III	12	29	Positive	Positive
10	Yes	III	35	56	Negative	Negative
11	Yes	III	45	55	Negative	Negative
12	No	III	3	7	Positive	Positive
13	No	IIA	54	54	Negative	Negative
14	Yes	IIA	54	54	Negative	Negative
15	No	III	1	27	Positive	Negative
16	Yes	IV	5	53	Positive	Positive
17	Yes	IIA	52	52	Positive	Positive
18	Yes	IIA	51	51	Positive	Positive
19	Yes	IIA	50	50	Negative	Negative
20	Yes	IIA	49	49	Negative	Negative
21	Yes	III	49	49	Negative	Negative
22	Yes	IV	6	49	Negative	Negative
23	Yes	IIB	48	48	Negative	Negative
24	No	IIA	3	24	Positive	Positive
25	Yes	III	33	47	Negative	Negative
26	Yes	IIA	46	46	Negative	Negative
27	Yes	IIA	46	46	Negative	Negative
28	No	III	9	10	Positive	Positive
29	Yes	III	46	46	Negative	Negative
30	No	III	2	46	Positive	Negative
31	Yes	III	45	45	Negative	Negative
32	Yes	IIA	45	45	Negative	Negative
33	No	IIB	45	45	Positive	Positive
34	No	III	44	44	Negative	Negative
35	No	III	13	13	Negative	Positive
36	No	III	16	16	Positive	Negative
37	No	III	10	42	Negative	Negative
38	Yes	IIA	42	42	Negative	Negative
39	Yes	IIA	42	42	Negative	Negative
40	Yes	IIA	22	42	Negative	Negative

Patients	Genter	Age	Location	Operation type	Tumor necrosis rate (%)	Recurrence
41	female	35	Proximal humerus	Limb salvage	≥90	No
42	female	18	Proximal tibia	Limb salvage	< 90	Yes
43	male	10	Distal femur	Limb salvage	< 90	No
44	male	34	Proximal tibia	Limb salvage	< 90	No
45	female	15	Distal femur	Limb salvage	< 90	Yes
46	female	14	Distal femur	Limb salvage	< 90	Yes
47	male	27	Proximal femur	Limb salvage	< 90	No
48	female	55	Distal femur	Amputation	< 90	No
49	male	18	Proximal humerus	Limb salvage	≥90	No
50	male	17	Distal femur	Limb salvage	≥90	No
51	female	33	Distal femur	Limb salvage	< 90	No
52	female	19	Proximal femur	Limb salvage	< 90	No
53	female	21	Proximal femur	Limb salvage	< 90	No
54	male	15	Proximal tibia	Limb salvage	< 90	No
55	female	16	Distal femur	Limb salvage	≥90	No
56	female	11	Distal femur	Limb salvage	< 90	No
57	female	31	Proximal humerus	Limb salvage	< 90	No
58	male	23	Proximal humerus	Limb salvage	< 90	No
59	male	23	Distal femur	Limb salvage	≥90	No
60	female	14	Distal femur	Limb salvage	< 90	No
61	female	38	Proximal femur	Limb salvage	< 90	No
62	female	56	Proximal tibia	Limb salvage	≥90	No
63	female	8	Distal femur	Limb salvage	< 90	No
64	female	50	Distal femur	Amputation	< 90	No
65	male	12	Distal femur	Limb salvage	≥90	No
66	female	19	Distal femur	Limb salvage	< 90	No
67	female	7	Distal femur	Limb salvage	≥90	No
68	male	19	Distal femur	Limb salvage	≥90	No
69	female	55	Proximal femur	Limb salvage	< 90	No
70	female	24	Distal femur	Limb salvage	< 90	No
71	female	14	Distal femur	Limb salvage	< 90	No
72	male	12	Distal femur	Limb salvage	< 90	No
73	female	14	Distal femur	Limb salvage	< 90	No
74	female	21	Proximal femur	Limb salvage	< 90	No
75	female	9	Distal femur	Limb salvage	< 90	No
76	female	34	Distal femur	Limb salvage	< 90	No
77	female	13	Distal femur	Limb salvage	≥90	No
78	male	18	Proximal femur	Limb salvage	≥90	No
79	male	17	Distal femur	Amputation	≥90	No
80	female	11	Distal femur	Limb salvage	< 90	Yes

Patients	Metastasis	AJCC/TNM stage	Tumor-Free Survival (Month)	Overall Survival (Month)	CLTC expression	TFG expression
41	No	IIA	42	42	Negative	Negative
42	Yes	III	16	16	Negative	Negative
43	No	IIA	41	41	Negative	Negative
44	No	III	15	15	Negative	Positive
45	No	IIB	7	8	Positive	Positive
46	No	III	10	41	Negative	Negative
47	Yes	IIA	40	40	Negative	Negative
48	Yes	III	40	40	Negative	Positive
49	Yes	IIA	40	40	Negative	Negative
50	Yes	III	39	39	Negative	Negative
51	No	III	39	39	Negative	Positive
52	Yes	III	32	32	Positive	Positive
53	Yes	IIA	39	39	Negative	Negative
54	Yes	IIA	39	39	Negative	Negative
55	No	IIA	39	39	Negative	Negative
56	No	IIB	38	38	Positive	Positive
57	Yes	IIA	41	41	Negative	Negative
58	No	III	40	40	Negative	Negative
59	Yes	IIA	38	38	Negative	Negative
60	Yes	III	38	38	Negative	Negative
61	No	III	38	38	Negative	Negative
62	No	III	8	38	Positive	Positive
63	No	IIA	38	38	Negative	Negative
64	No	IIA	37	37	Negative	Negative
65	No	III	17	64	Negative	Negative
66	No	III	6	37	Positive	Positive
67	Yes	IIA	37	37	Negative	Negative
68	Yes	III	37	37	Negative	Negative
69	Yes	IIB	37	37	Negative	Negative
70	Yes	IIA	6	37	Negative	Negative
71	Yes	IIA	36	36	Positive	Positive
72	No	III	4	36	Positive	Positive
73	No	IIA	36	36	Negative	Negative
74	Yes	III	16	53	Negative	Negative
75	No	III	5	35	Negative	Negative
76	No	IIB	35	35	Negative	Negative
77	Yes	IV	34	34	Negative	Negative
78	No	III	4	34	Positive	Positive
79	Yes	III	34	34	Positive	Positive
80	No	III	10	18	Negative	Negative

Patients	Genter	Age	Location	Operation type	Tumor necrosis rate (%)	Recurrence
81	male	15	Distal femur	Limb salvage	< 90	No
82	male	57	Proximal tibia	Limb salvage	< 90	No
83	female	32	Proximal tibia	Amputation	< 90	No
84	female	14	Distal femur	Limb salvage	< 90	Yes
85	male	16	Distal femur	Amputation	< 90	No
86	male	9	Proximal tibia	Limb salvage	≥90	No
87	male	12	Distal radius	Amputation	< 90	No
88	female	28	Proximal tibia	Limb salvage	< 90	No
89	female	13	Proximal tibia	Limb salvage	< 90	No
90	female	13	Distal femur	Limb salvage	< 90	No
91	male	25	Proximal femur	Limb salvage	≥90	No
92	female	23	Distal femur	Limb salvage	< 90	No
93	female	15	Distal femur	Limb salvage	< 90	No
94	female	23	Proximal femur	Limb salvage	≥90	No
95	female	17	Distal femur	Limb salvage	< 90	Yes
96	female	13	Proximal tibia	Limb salvage	< 90	No
97	female	12	Distal femur	Limb salvage	< 90	Yes
98	male	21	Distal femur	Limb salvage	≥90	No
99	female	10	Distal femur	Limb salvage	< 90	Yes
100	male	29	Proximal tibia	Limb salvage	< 90	No
101	male	36	Distal femur	Limb salvage	≥90	No
102	male	34	Proximal tibia	Limb salvage	< 90	No

Patients	Metastasis	AJCC/TNM stage	Tumor-Free Survival (Month)	Overall Survival (Month)	CLTC expression	TFG expression
81	No	III	33	33	Negative	Negative
82	No	IIA	33	33	Negative	Negative
83	Yes	IIA	33	33	Negative	Negative
84	No	III	12	12	Negative	Negative
85	No	IIB	33	33	Positive	Positive
86	No	III	32	32	Positive	Positive
87	No	IIA	32	32	Negative	Negative
88	No	IIA	31	31	Negative	Negative
89	No	IIA	31	31	Negative	Negative
90	No	IIA	31	31	Negative	Positive
91	No	IIA	51	51	Negative	Negative
92	No	IIA	36	36	Negative	Negative
93	No	IIA	44	44	Negative	Negative
94	Yes	IIA	53	53	Negative	Positive

95	No	IIB	12	21	Negative	Negative
96	No	IIA	19	68	Negative	Negative
97	No	IIB	21	21	Negative	Negative
98	Yes	IIA	51	51	Negative	Negative
99	No	III	11	41	Positive	Positive
100	No	III	30	30	Negative	Negative
101	Yes	IIA	51	51	Negative	Negative
102	No	III	30	30	Negative	Negative
